# Concomitant Administration of Dantrolene is Sufficient to Protect Against Doxorubicin-Induced Cardiomyopathy

**DOI:** 10.1016/j.jaccao.2024.10.011

**Published:** 2024-12-10

**Authors:** Yoshihide Nakamura, Takeshi Yamamoto, Shigeki Kobayashi, Takeshi Suetomi, Hitoshi Uchinoumi, Tetsuro Oda, Motoaki Sano, Masafumi Yano

**Affiliations:** a Department of Therapeutic Science for Heart Failure in the Elderly, Yamaguchi University Graduate School of Medicine, Ube, Japan; b Faculty of Health Sciences, Yamaguchi University Graduate School of Medicine, Ube, Japan; c Department of Medicine and Clinical Science, Division of Cardiology, Yamaguchi University Graduate School of Medicine, Ube, Japan

**Keywords:** anthracycline, Ca^2+^, calmodulin, cardiomyopathy, doxorubicin, heart failure, mechanisms, ryanodine receptor

## Abstract

**Background:**

Doxorubicin (DOX), a commonly used anticancer agent, can result in cardiac dysfunction, presenting a significant clinical challenge. DOX has been shown to induces Ca^2+^ leakage via the ryanodine receptor 2 (RYR2) of the sarcoplasmic reticulum, increasing Ca^2+^ levels in the cytoplasm.

**Objectives:**

This study investigated whether stabilizing RYR2 could suppress DOX-induced cardiomyopathy (DIC) and identified the optimal duration of dantrolene treatment as a pharmacological method.

**Methods:**

We investigated the effects of RYR2 stabilization on DOX cardiotoxicity using in vivo and in vitro experiments.

**Results:**

DOX administration caused calmodulin dissociation, marked Ca^2+^ leakage from RYR2, and increased oxidative stress in isolated cardiomyocytes. Stabilizing the RYR2 tetramer—either pharmacologically with dantrolene or genetically via RYR2 V3599K mutation, which enhances calmodulin binding affinity—suppressed these effects. In DIC mice models, DOX impaired cardiac function, increased fibrosis and TUNEL-positive cells, reduced GRP78, and elevated lipid peroxide levels, leading to endoplasmic reticulum stress and ferroptosis. Both continuous dantrolene treatment and RYR2 V3599K mutation improved cardiac function. Interestingly, dantrolene administration provided myocardial protection even when terminated 7 days after DOX.

**Conclusions:**

Short-term concomitant use of dantrolene offers a promising and clinically feasible strategy to prevent DIC. Given dantrolene’s established clinical safety as a treatment for malignant hyperthermia, these findings suggest potential for repositioning dantrolene in DIC prevention.

Advances in chemotherapy have significantly improved cancer patient outcomes; however, the side effects of many anticancer drugs can diminish prognosis and quality of life.^1^ In particular, doxorubicin (DOX), an anthracycline with strong antitumor effects, is essential for many cancer treatments but is also associated with significant cardiotoxicity.^2^ Heart failure can result in treatment interruptions, and the resulting cardiac dysfunction from DOX can be irreversible, posing a major clinical problem.^3^ DOX-induced heart damage involves several mechanisms, including mitochondrial damage,^4^ reactive oxygen species (ROS) production,^1^ intracellular calcium (Ca^2+^) overload,^5^^,^^6^ and endoplasmic reticulum (ER) stress.^7^

Notably, Ca^2+^ leakage via ryanodine receptor 2 (RYR2), a Ca^2+^ release channel on the sarcoplasmic reticulum (SR), significantly affects cardiac dysfunction.^8^ Research has shown that RYR2-mediated Ca^2+^ leakage in heart failure and catecholamine polymorphic ventricular tachycardia occurs when the channel becomes unstable due to impaired associations between its N-terminal and central domains.^8^^,^^9^ Specifically, defective interdomain interactions within RYR2 cause calmodulin (CaM) to dissociate from the receptor, destabilizing it and leading to Ca^2+^ leakage. We previously reported that dantrolene (DAN), a stabilizer of RYR2, binds directly to the N-terminal domain of RYR2, enhancing CaM binding affinity and stabilizing the channel, thereby preventing Ca^2+^ leakage.^10^

Recently, we developed a mouse model with a single amino acid mutation in the CaM binding domain of RYR2 (V3599K), which strengthens CaM binding affinity. In RYR2 V3599K knock-in (KI) mice crossed with catecholamine polymorphic ventricular tachycardia–type KI mice, abnormal Ca^2+^ leakage was eliminated, and ventricular tachycardia was completely suppressed.^11^ Furthermore, we found that ER stress—resulting from destabilization of the RYR2 tetramer structure, CaM dissociation, and Ca^2+^ leakage—contributes to disease in other organs. Pharmacologically or genetically preventing CaM dissociation from RyR2 showed protective effects against fatty liver and Alzheimer’s disease.^12^^,^^13^

Conversely, studies have shown that DOX can bind RYR2, contributing to Ca^2+^ leakage from the SR and increasing cytoplasmic Ca^2+^ levesl,^14^^,^^15^ and that DAN may be effective against doxorubicin-induced cardiomyopathy (DIC).^16^^,^^17^ However, the underlying mechanisms remain unclear. Hence, we hypothesized that many of the mechanisms underlying DIC may involve CaM dissociation and Ca^2+^ leakage caused by RYR2 destabilization. Thus, this study aimed to determine whether RYR2 stabilization could prevent DIC and improve prognosis, while also elucidating the underlying mechanism of action. In addition, we assessed the optimal duration of DAN treatment.

## Methods

A detailed description of the methods is provided in the Supplemental Appendix. This study conformed to the *Guide for the Care and Use of Laboratory Animals* (National Academies Press). All animal care and protocols were approved by the Animal Ethics Committee of Yamaguchi University Graduate School of Medicine. Male and female C57BL/6 mice were used to generate DOX-induced cardiomyopathy models, following previously reported protocols with some modifications.^18^^,^^19^ Mice were anesthetized with isoflurane, and cardiac function was analyzed using an F37 ultrasound machine (Hitachi Medical) equipped with a 7.5-MHz probe (Hitachi, UST-5413).

Cardiomyocytes were isolated from mouse hearts as described previously.^20^ Intracellular Ca^2+^ dynamics were monitored using a laser-scanning confocal microscope (LSM-510, Carl Zeiss), following established protocols.^20^ Monitoring of intracellular ROS formation was assessed using the fluorescent probe, 2′,7′-dichlorofluorescin diacetate (DCFH-DA) (Thermo Fisher Scientific).

Immunocytochemistry was performed as described previously.^11^ Mitochondrial lipid peroxidation was assessed by measuring lipid peroxides in the inner mitochondrial membrane using MitoPeDPP (DOJINDO Laboratories). The extent of fibrosis was analyzed with Picro-Sirius Red staining (PSR-1, ScyTek Laboratories). Intracellular DNA fragments were detected via terminal deoxynucleotidyl transferase dUTP nick end labeling (TUNEL) staining using the In Situ Cell Death Detection Kit, Fluorescein (Roche).

### Statistical analysis

Two-tailed Student’s *t*-tests were used for comparisons between 2 conditions, whereas 1-way analysis of variance with post hoc Tukey’s or Dunnett’s test was applied for comparisons among multiple groups. A repeated measures analysis of variance controlled for within-mouse correlations. Data normality was determined using the Shapiro-Wilk test in JMP pro 16 software (JMP Statistical Discovery). All other statistical analysis and plotting were performed using GraphPad Prism 5 software (GraphPad Software).

All data are expressed as mean ± SEM. Statistical significance was set at *P* < 0.05. Kaplan-Meier plots were generated for survival analysis in each group, with significance determined by log-rank tests.

## Results

### RYR2 stabilization suppresses DOX-induced Ca^2+^ leakage and ROS production

First, we measured Ca^2+^ sparks, which indicate Ca^2+^ leakage from RYR2, in isolated cardiomyocytes exposed to varying concentrations of DOX. Ca^2+^ spark frequency (SpF) significantly increased within 5 minutes after treatment with 1 μmol/L or 3 μmol/L DOX. This increase was suppressed by either DAN treatment or the RYR2 V3599K mutation, regardless of DOX concentration used (Supplemental Figure 1).

**Figure 1:**
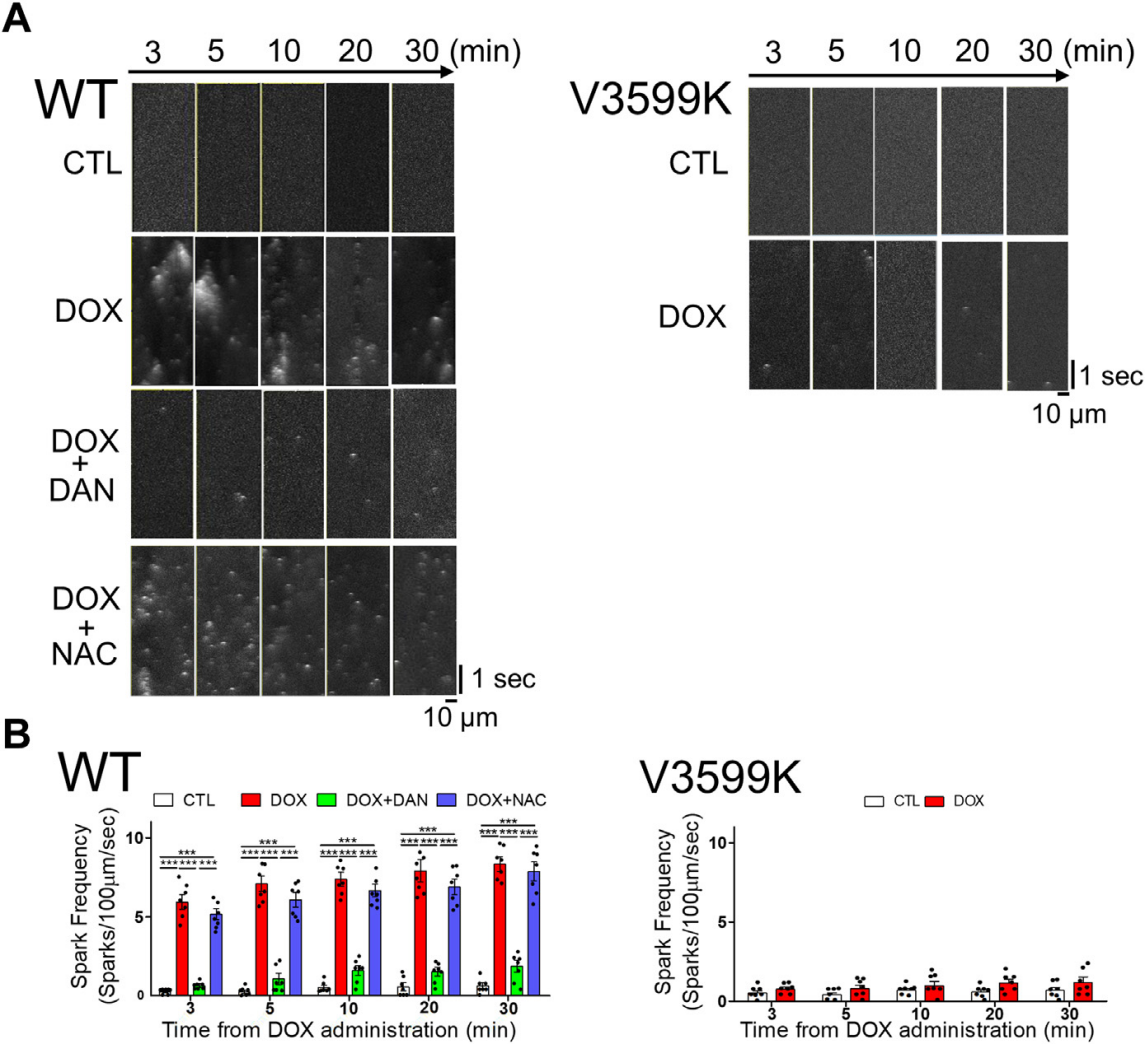
DOX-Induced Ca^2+^ Spark Increase Is Suppressed by RYR2 Stabilization (A) Representative line scan images of Fluo-4 AM fluorescence in intact wild-type (WT) and ryanodine receptor 2 (RYR2) V3599K-KI cardiomyocytes. (B) Data summary. Control (CTL): no doxorubicin (DOX); DOX: 1 μmol/L; dantrolene (DAN): 1 μmol/L; *N*-acetyl cysteine (NAC): 1 mmol/L. Mean ± SEM (n = 50-66 cells from 7 hearts). ∗∗∗*P* < 0.001 by 1-way analysis of variance with Tukey's post hoc test. KI = knock-in.

Previous reports have shown that DOX chemotherapy patients reach peak plasma concentrations of 0.5 to 1 μmol/L.^21^^,^^22^ Therefore, we monitored Ca^2+^ sparks over time after the addition of DOX at this fixed 1 μmol/L concentration. Ca^2+^ SpF increased markedly from soon after DOX exposure and continued to rise. This DOX-induced Ca^2+^ leakage was suppressed by DAN treatment or in RYR2 V3599K-KI cardiomyocytes but was unaffected by *N*-acetyl cysteine (NAC) treatment, suggesting that DOX-induced Ca^2+^ leakage is mediated directly through RYR2 rather than through ROS (Figure 1).

To elucidate the underlying mechanism of DOX-induced Ca^2+^ leakage from RYR2, we evaluated the relationship between Ca^2+^ SpF and SR Ca^2+^ content in saponin-permeabilized cardiomyocytes. In wild-type (WT) cardiomyocytes, DOX administration caused a significant leftward shift in the SpF vs SR Ca^2+^-content curve. Interestingly, DAN shifted this relationship back to the right, nearly to the control level. Similarly, in RYR2 V3599K-KI cardiomyocytes, the SpF/SR Ca^2+^-content relationship remained stable despite DOX exposure (Supplemental Figure 2).

**Figure 2:**
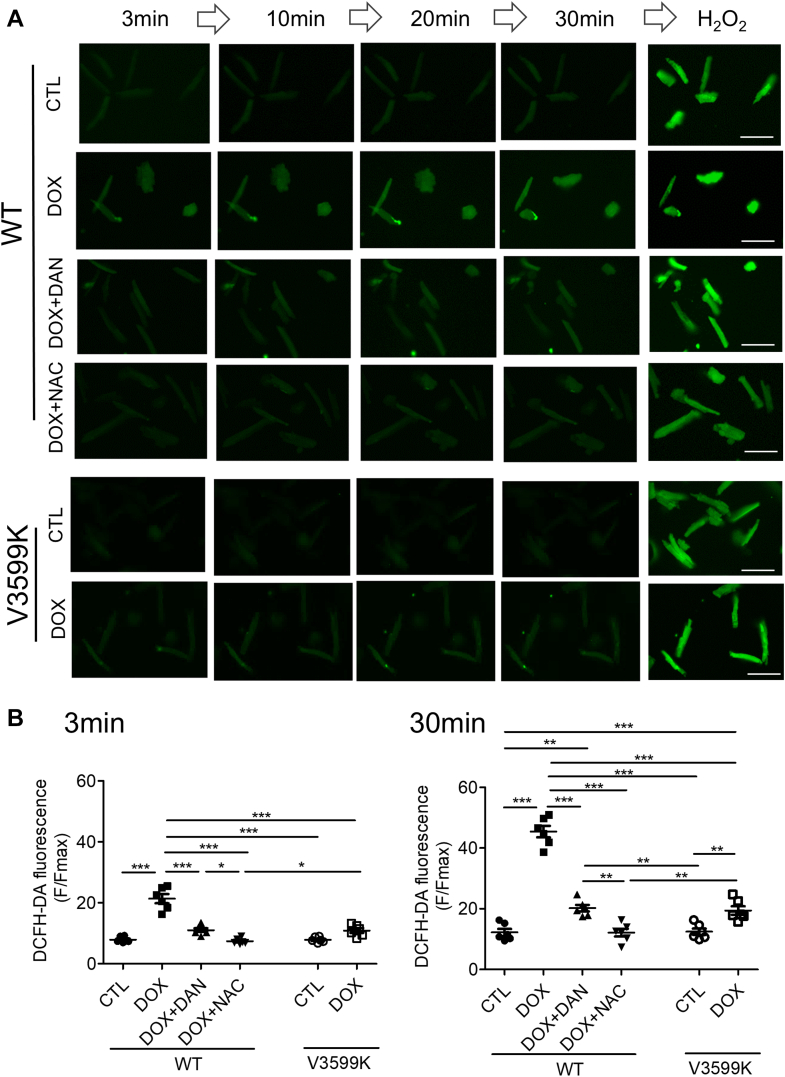
RYR2 Stabilization Suppresses DOX-Induced Oxidative Stress (A) Representative images of DCFH-DA fluorescence in intact WT and RYR2 V3599K-KI cardiomyocytes. (B) Data summary. Scale bar: 100 μm. CTL: no DOX; DOX: 1 μmol/L; DAN: 1 μmol/L; NAC: 1 mmol/L. Mean ± SEM (n = 113-155 cells from 6 hearts). ∗*P* < 0.05, ∗∗*P* < 0.01, and ∗∗∗*P* < 0.001 by 1-way analysis of variance with Tukey's post hoc test. Abbreviations as in Figure 1.

Next, we assessed DOX- induced ROS levels. DOX was added to WT cardiomyocytes at various concentrations, and ROS levels were measured using DCFH-DA after 24 hours of culture. ROS levels significantly increased at DOX concentrations above 1 μmol/L (Supplemental Figure 3). When tracking ROS over time after 1 μmol/L DOX administration, ROS levels elevated shortly after DOX administration and continued to gradually rise. Interestingly, this ROS elevation was suppressed by either DAN treatment or the RYR2 V3599K mutation (Figure 2), suggesting that ROS production induced by therapeutic DOX concentrations is mediated by Ca^2+^ leakage through RYR2.

### Enhanced CaM binding to RYR2 prevents DOX-induced decrease in sarcomere shortening

**Figure 3:**
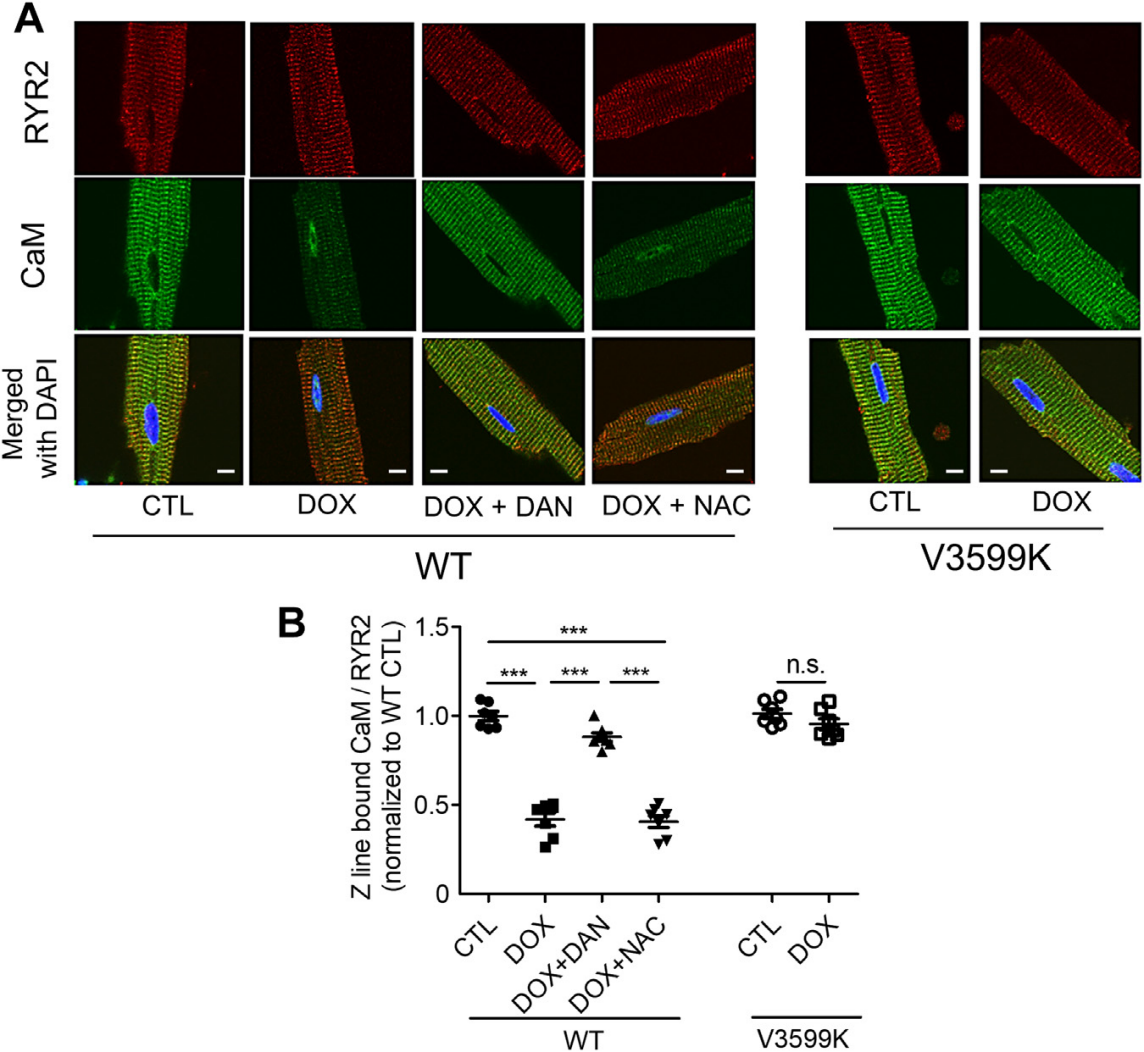
RYR2 Stabilization Maintains RYR2-Bound CaM Despite DOX-Induced Dissociation (A) Representative images of endogenous calmodulin (CaM) and RYR2 in intact WT and RYR2 V3599K-KI cardiomyocytes fixed 5 minutes after adding DOX (1 μmol/L), DOX (1 μmol/L) + DAN (1 μmol/L), or DOX (1 μmol/L) + NAC (1 mmol/L). RYR2 is shown in red; CaM is shown in green. (B) Data summary. Scale bar: 10 μm. Mean ± SEM (n = 45-62 cells from 7 hearts); ∗∗∗*P* < 0.001 by 1-way analysis of variance with Tukey's post hoc test; not significant (n.s.) by unpaired Student’s *t*-test. Abbreviations as in Figure 1.

We evaluated RYR2 and CaM fluorescence in cardiomyocytes using immunostaining 5 minutes after the addition of 1 μmol/L DOX (Figure 3). As previously reported,^11^ CaM was well colocalized with RYR2 along the Z line. After DOX exposure, RYR2-bound CaM fluorescence markedly decreased. However, with DAN treatment or the RYR2 V3599K mutation (but not NAC), CaM fluorescence remained stable despite the presence of DOX.

**Figure 4:**
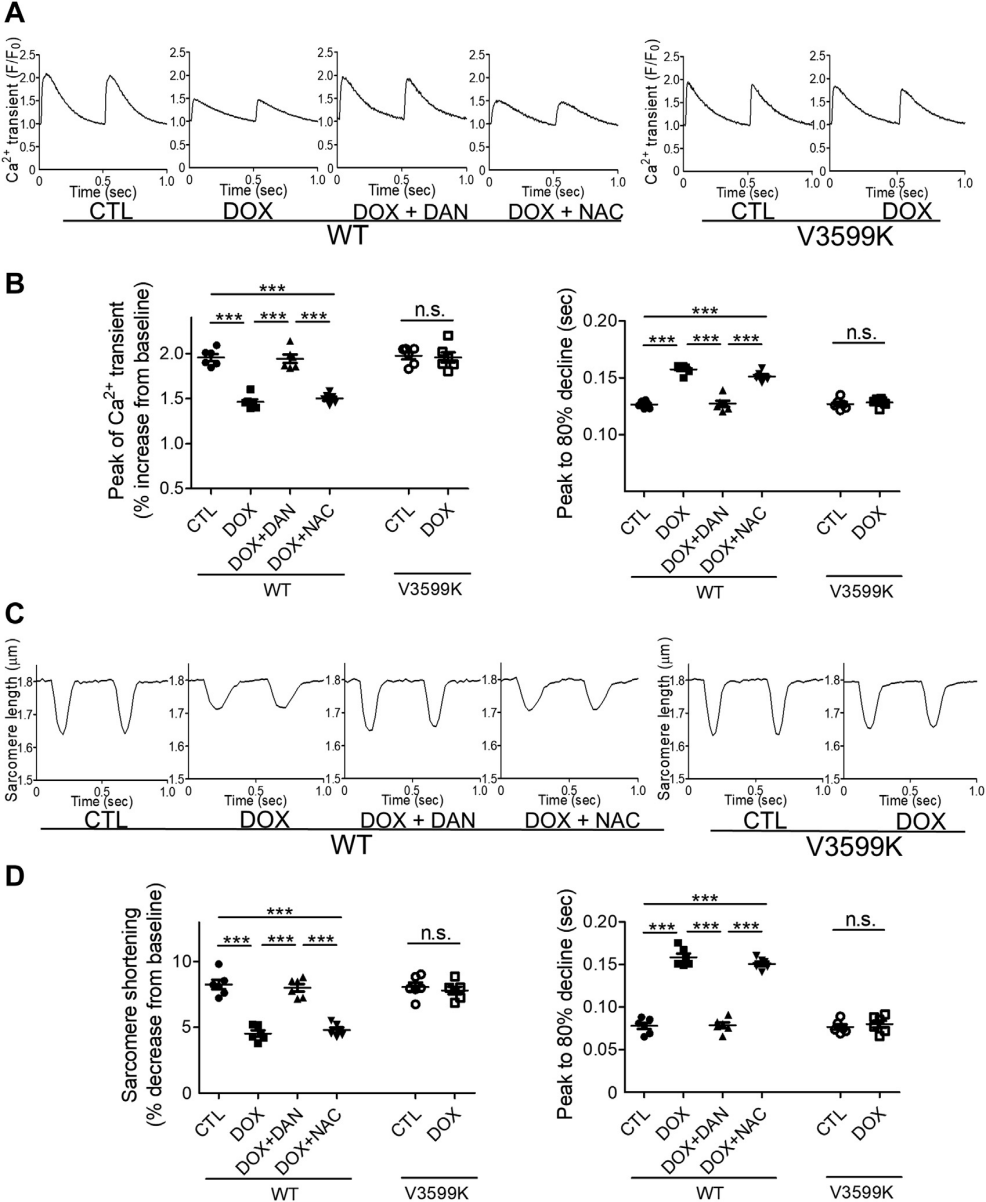
Effects of DOX on Ca^2+^ Transient and Sarcomere Shortening (A) Representative recordings of Fluo-4 AM fluorescence signals at 2-Hz pacing in intact WT and RYR2 V3599K-KI cardiomyocytes 30 minutes after observation. (B) Data summary (mean ± SEM; n = 34-42 cells from 6 hearts). (C) Representative recordings of sarcomere length at 2-Hz pacing in intact WT and RYR2 V3599K-KI cardiomyocytes 30 minutes after observation. (D) Data summary. CTL: no DOX; DOX: 1 μmol/L; DAN: 1 μmol/L; NAC: 1 mmol/L. Mean ± SEM (n = 35-47 cells from 6 hearts). ∗∗∗*P* < 0.001 by 1-way analysis of variance with Tukey's post hoc test; n.s. by unpaired Student’s *t*-test. F/F_0_ = fluorescent intensity/baseline fluorescent intensity; other abbreviations as in Figures 1 and 3.

We observed Ca^2+^ transients and sarcomere shortening 30 minutes after DOX administration (Figure 4). DOX reduced both the peak of the Ca^2+^ transient and sarcomere shortening while also prolonging the time required for an 80% decline from the peak. These findings suggest that these changes are mediated by Ca^2+^ leakage through RYR2 and subsequent Ca^2+^ depletion in the SR, rather than from oxidative stress, as they were mitigated by RYR2 stabilization, but not by antioxidation.

### DOX does not accumulate in cardiomyocytes

DOX is known to exhibit fluorescence,^23^ which we used to evaluate its binding to crude heart homogenates and SR vesicles using the DOX-SMCC (sDOX), an agent-linker conjugate for antibody-drug conjugate. sDOX fluorescence was observed at various levels in crude heart homogenates (Supplemental Figure 4) and aligned with RYR2 in SR vesicles (Supplemental Figure 5A).

**Figure 5:**
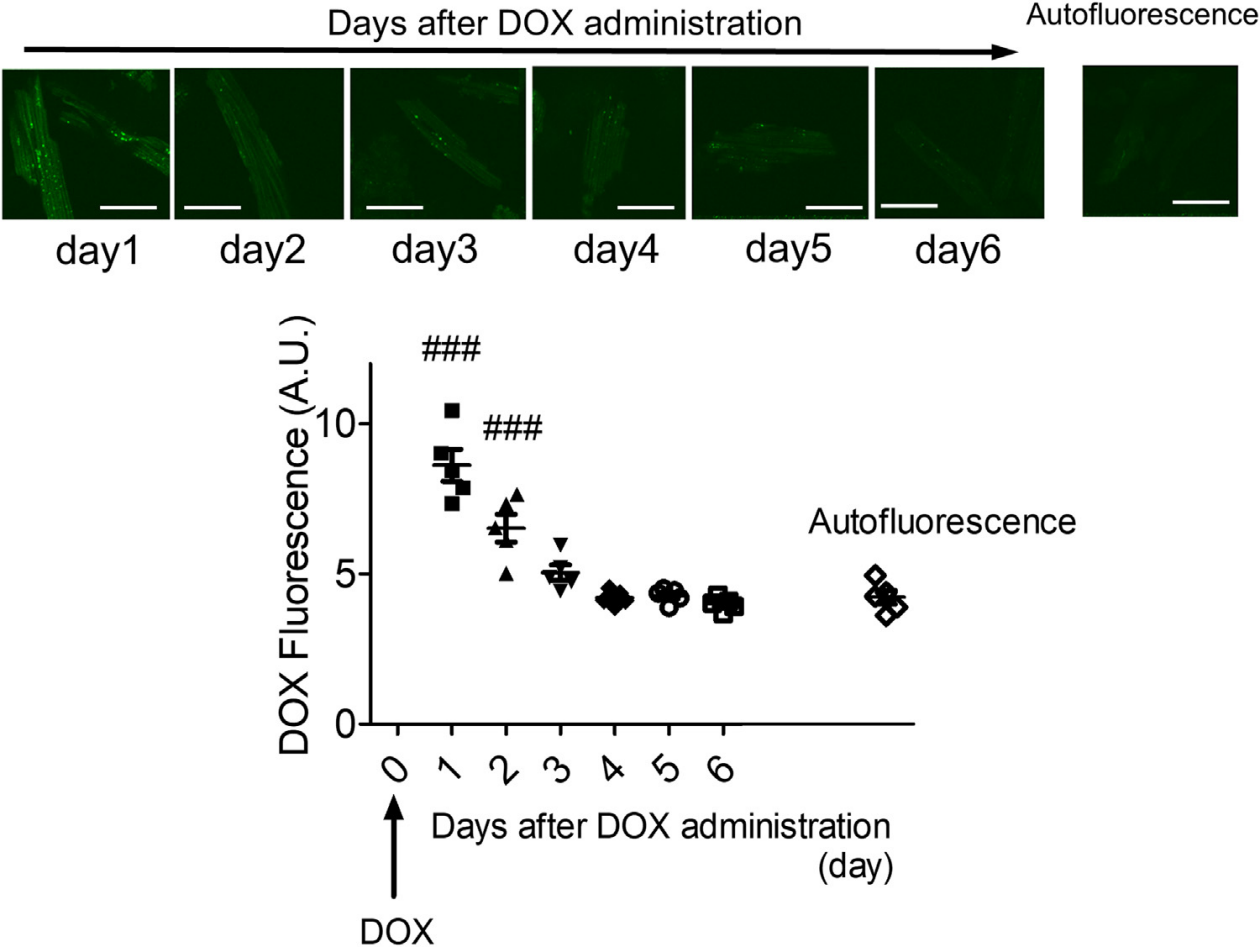
Intracellular DOX Fluorescence Over Time Intracellular DOX fluorescence assessed in isolated cardiomyocytes from different mice at each time point after DOX administration. Scale bar: 50 μm. Mean ± SEM (n = 30-35 cells from 5 hearts); ###*P* < 0.001 vs autofluorescence by 1-way analysis of variance with Dunnett’s post hoc test. Abbreviations as in Figure 1.

Moreover, we confirmed sDOX binding sites within RYR2 using recombinant RYR2 fragments, finding that sDOX specifically bound to fragment1-610 and fragment2234-2750 (Supplemental Figure 5B). However, no DOX fluorescence was observed inside isolated cardiomyocytes evaluated 4 weeks after the last DOX injection in the DIC mouse model (data not shown). To determine how long DOX fluorescence persists in cardiomyocytes after DOX administration, we administered a single dose of DOX (6 mg/kg) intraperitoneally and measured cardiomyocyte fluorescence daily. DOX fluorescence decreased progressively and was indistinguishable from autofluorescence by the third day after injection (Figure 5). These findings indicate that although DOX exhibits cardiotoxicity, it does not accumulate intracellularly.

### Stabilizing RYR2 maintains cardiac function despite DOX exposure

Based on previous results, we hypothesized that DAN could provide effective myocardial protection during and shortly after DOX administration. To prove this, we first examined survival rates after a single intraperitoneal DOX dose (24 mg/kg) in mice. All WT mice treated with DOX (WT-DOX) died within 50 days, whereas survival was significantly improved in mice receiving continuous DAN (WT-DOX+DAN). Similarly, survival rates were markedly improved in RYR2 V3599K-KI mice (V3599K-DOX). Surprisingly, even when DAN was terminated 7 days after DOX administration (WT-DOX+DAN [short]), survival was comparable to that of mice with continuous DAN administration (Supplemental Figure 6A).

At 20 days after DOX administration, although body weight remained stable across all groups, cardiac function decreased in the WT-DOX group but was preserved in the WT-DOX+DAN, V3599K-DOX, and WT-DOX+DAN (short) groups. In the long-term follow-up study, survival gradually decreased even in groups other than WT-DOX, likely due to severe systemic (noncardiotoxic) organ damage from the very high DOX doses, which may exceed typical clinical levels. Supporting this, 2 V3599K-DOX mice that survived for 300 days displayed significant weight loss but maintained well-preserved cardiac function (Supplemental Figures 6B to 6D).

**Figure 6:**
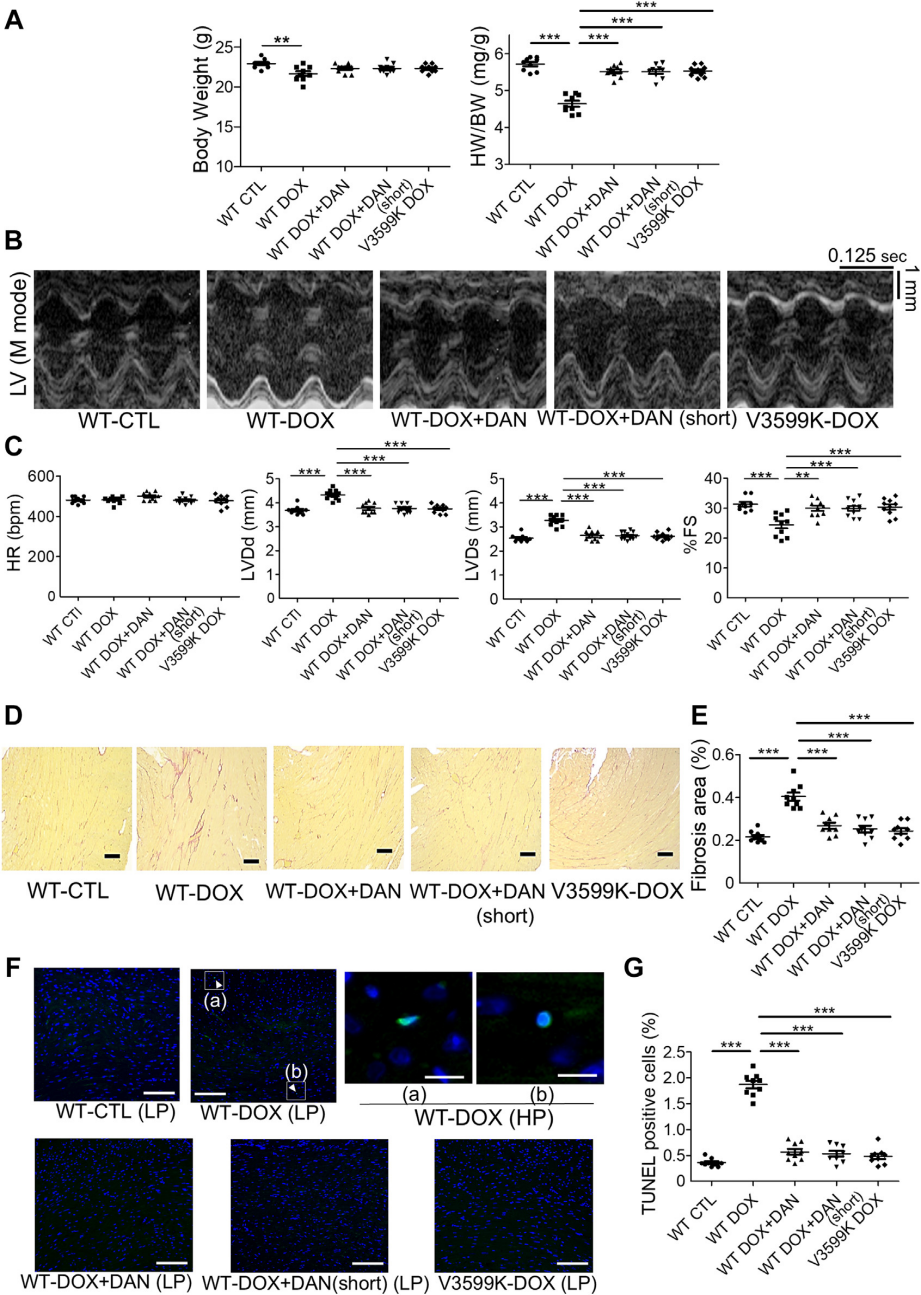
DAN Prevents DOX-Induced Cardiac Dysfunction in Chronic Model Mice (A) Body weight (BW) and heart weight (HW) normalized to BW (n = 9). (B) Echocardiographic images and (C) summary of data (n = 9-11). (D) Collagen fibers in the left ventricle (LV) evaluated by Picro-Sirius Red staining and (E) data summary. Collagen is shown in red; muscle fibers in yellow (n = 9). Scale bar: 100 μm. (F) TUNEL staining images at low power (LP) (scale bar: 100 μm) and high power (HP) (scale bar: 10 μm) fields, and (G) data summary (n = 9). Arrowheads indicate TUNEL-positive nuclei. Data acquired as shown in Supplemental Figure 7A. ∗∗*P* < 0.01 and ∗∗∗*P* < 0.001 by 1-way analysis of variance with Tukey's post hoc test. Values for individual mice are plotted as mean ± SEM. bpm = beats/min; HR = heart rate; other abbreviations as in Figure 1.

Next, we examined the stabilizing effects of RYR2 on body weight, cardiac morphology, and cardiac function in DIC model mice 4 weeks after the final DOX administration (at 16 weeks of age) (Supplemental Figure 7A). Body weight, heart weight/body weight, left ventricular mass on echocardiography, and cardiomyocyte size in histology sections were all decreased in the WT-DOX group but were preserved in the WT-DOX+DAN (short), WT-DOX+DAN, and V3599K-DOX groups (Figure 6A, Supplemental Figures 8 and 9). Echocardiographic findings showed left ventricular end-diastolic diameter/end-systolic diameter was enlarged, and percentage fractional shortening decreased in the WT-DOX group starting from the DOX administration period. By contrast, cardiac function was maintained in the WT-DOX+DAN, V3599K-DOX, and WT-DOX+DAN (short) groups (Figures 6B and 6C, Supplemental Figure 10).

We found no sex differences in these findings (Supplemental Figure 11). Additionally, tissue fibrosis area and cell death were evaluated by Picro-Sirius Red and TUNEL staining, respectively. Both were elevated in the WT-DOX group but were significantly suppressed in the WT-DOX+DAN, V3599K-DOX, and WT-DOX+DAN (short) groups (Figures 6D to 6G).

**Figure 7:**
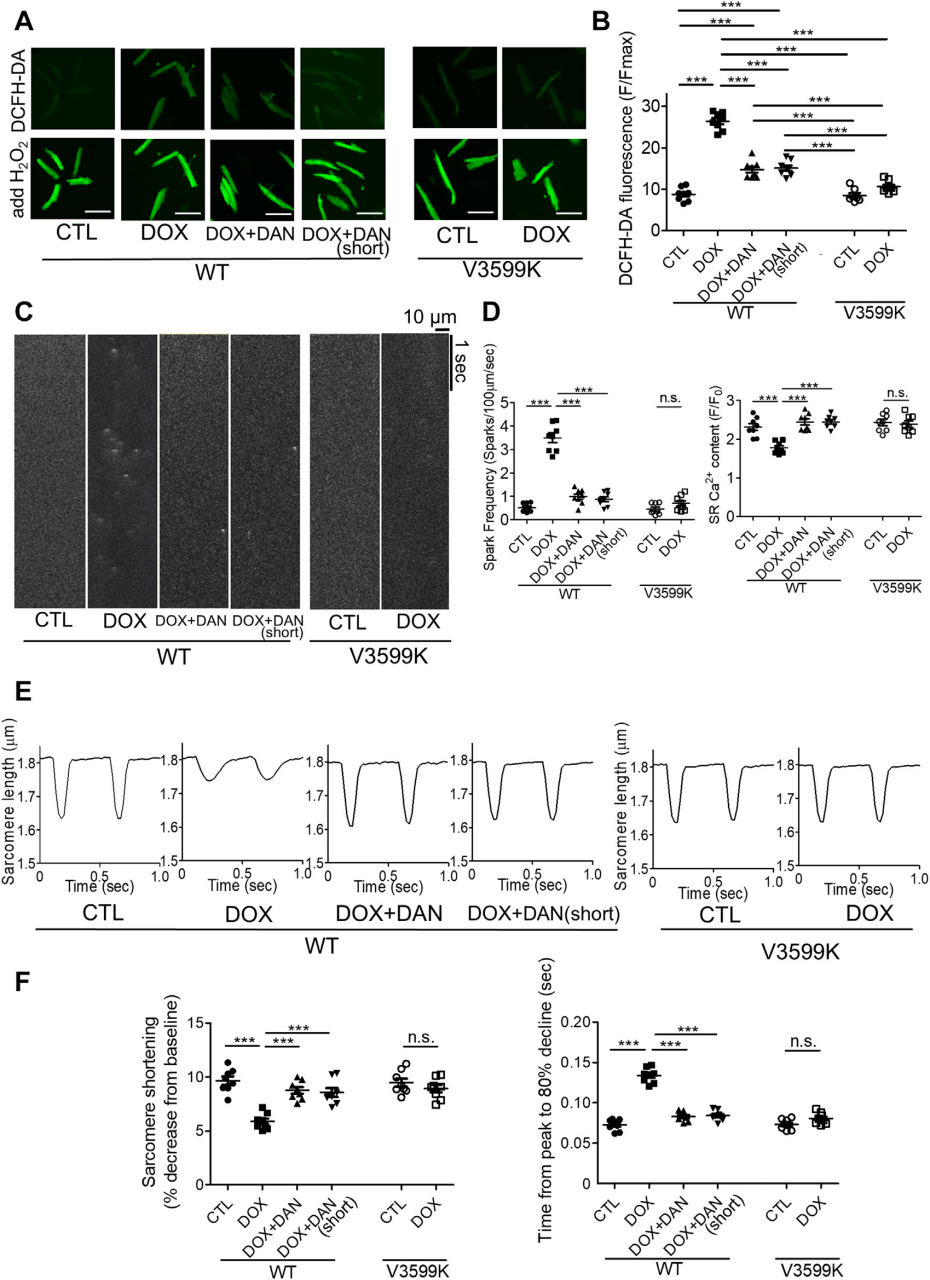
Evaluation of Isolated Cardiomyocytes in Chronic Model Mice (A) Representative images of DCFH-DA fluorescence in isolated cardiomyocytes and (B) summary of data. Scale bar: 100 μm. Mean ± SEM (n = 65-78 cells from 8 hearts). (C) Representative line scan images of Fluo-4 AM fluorescence in isolated cardiomyocytes and (D) data summary. Mean ± SEM (n = 42-63 cells from 8 hearts). (E) Representative recordings of sarcomere length at 2-Hz pacing in isolated cardiomyocytes and (F) data summary. Mean ± SEM (n = 43-59 cells from 8 hearts). Data acquired as shown in Supplemental Figure 7A. ∗∗∗*P* < 0.001 by 2-way analysis of variance with Tukey's post hoc test; n.s. by unpaired Student’s *t*-test. Abbreviations as in Figures 1, 3, and 4.

Finally, we examined the stabilizing effects of RYR2 on cardiomyocyte function. Cardiomyocytes were obtained from mice 4 weeks after the last DOX administration. Both ROS levels and Ca^2+^ SpF were increased in the WT-DOX group but were suppressed in the WT-DOX+DAN (short) group, WT-DOX+DAN, and V3599K-DOX groups (Figures 7A to 7D). Additionally, the reduction in SR Ca^2+^ content observed in the WT-DOX group was prevented in the WT-DOX+DAN, V3599K-DOX, and WT-DOX+DAN (short) groups (Figure 7D).

Regarding sarcomere length changes, sarcomere shortening was reduced, and the time from peak to 80% decline was prolonged in the WT-DOX group. However, these measures improved in the WT-DOX+DAN, V3599K-DOX, and WT-DOX+DAN (short) groups (Figures 7E and 7F). These results strongly suggest that myocardial protection by DAN is effective and sufficient when administered during and for a short period after DOX administration.

### DOX-induced ER stress is prevented by RYR2 stabilization

We investigated the effects of RYR2 stabilization on DOX-induced ER stress and apoptosis. First, GRP78 and XBP-1 levels were evaluated by immunostaining cardiomyocytes isolated from mice 5 days after DOX administration. Nuclear XBP-1 fluorescence was consistently low across all groups. Interestingly, GRP78 fluorescence, which decreased with DOX treatment, was restored in both DOX plus DAN-treated WT mice and DOX-treated RYR2 V3599K-KI mice (Supplemental Figure 12). These results suggest that the DOX-induced reduction in GRP78 expression can be reversed by RYR2 stabilization, without being mediated by XBP-1.

**Figure 8:**
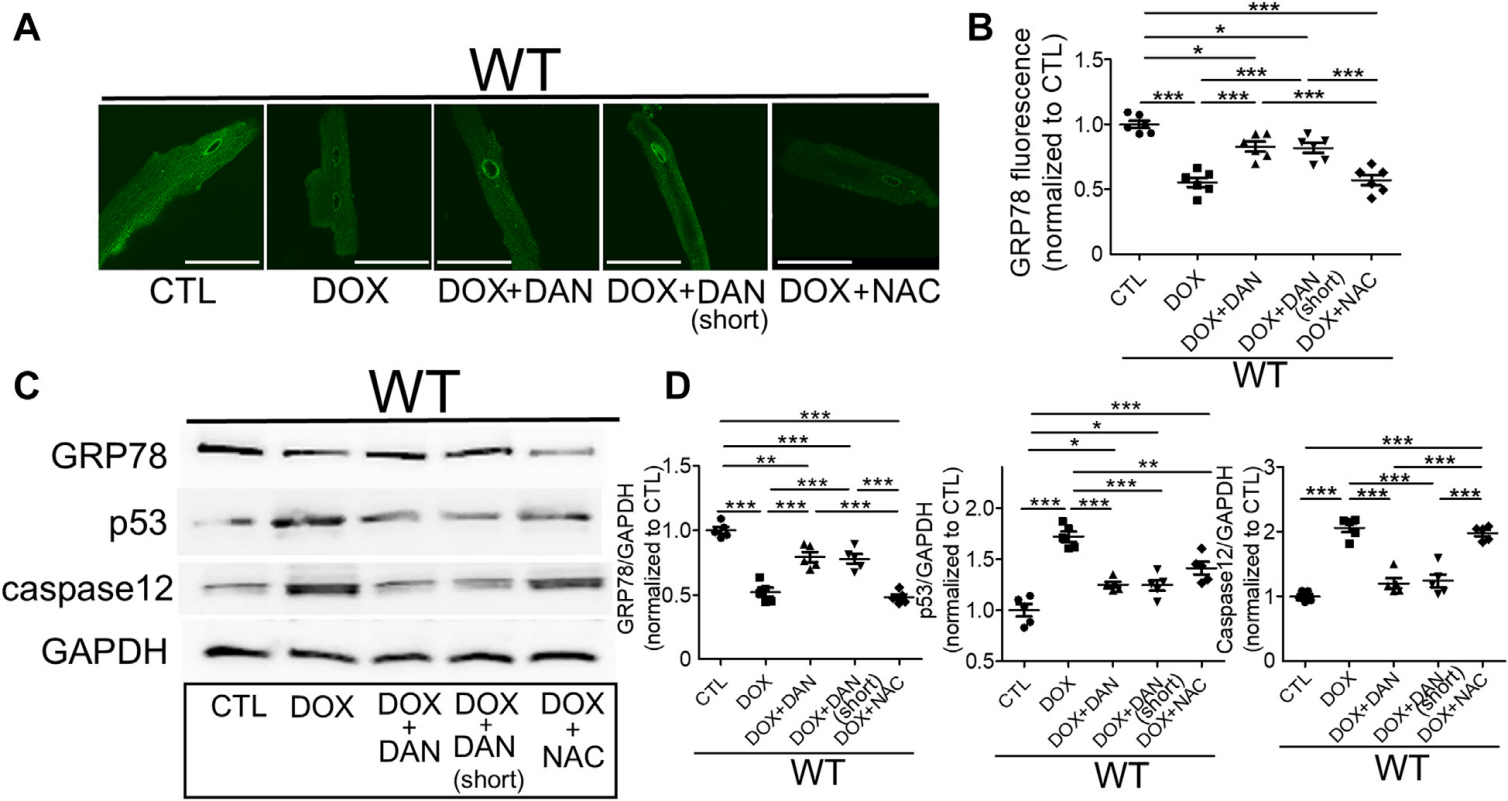
Evaluation of ER Stress Markers Changes in endoplasmic reticulum (ER) stress markers under control conditions or with DOX (1 μmol/L, 4 hours), DAN (1 μmol/L, 24 hours), DAN (short) (1 μmol/L, 4 hours), or NAC (1 mmol/L, 24 hours). (A) Representative immunostaining images of GRP78 and (B) data summary. Scale bar: 50 μm. Mean ± SEM (n = 52-58 cells from 6 hearts). (C) Western blots of GRP78, P53, caspase-12, and GAPDH in lysates from cardiomyocytes cultured for 24 hours and (D) data summary. Mean ± SEM (n = 5 hearts). ∗*P* < 0.05, ∗∗*P* < 0.01, and ∗∗∗*P* < 0.001 by 1-way analysis of variance with Tukey's post hoc test. Abbreviations as in Figure 1.

Next, isolated cardiomyocytes were treated as shown in Supplemental Figure 7B. In WT cardiomyocytes, GRP78 levels decreased, whereas caspase-12 and P53 levels increased in a DOX concentration–dependent manner (Supplemental Figures 13A to 13D). These effects were suppressed in RYR2 V3599K-KI cardiomyocytes, particularly at therapeutic DOX concentrations (Supplemental Figures 13E to 13H). When assessing the effects of DAN and NAC in WT cardiomyocytes at a fixed DOX concentration of 1 μmol/L, DOX-induced reductions in GRP78 levels were significantly reversed in both the DOX+DAN and DOX+DAN (short) groups, but not in the DOX+NAC group. Additionally, the DOX-induced increases in caspase-12 and P53 levels were suppressed in both the DOX+DAN (short) and DOX+DAN groups (Figure 8).

### DOX-induced ferroptosis is prevented by RYR2 stabilization

**Figure 9:**
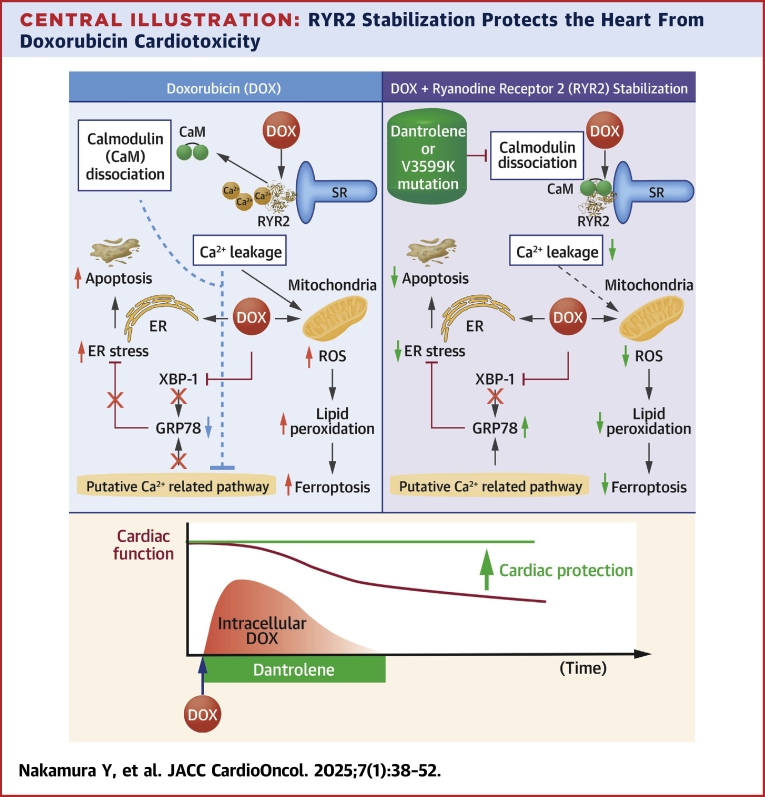
Evaluation of Ferroptosis Markers (A) Representative fluorescence images of mitochondrial lipid peroxidation in cultured cardiomyocytes under control conditions or with DOX (1 μmol/L, 4 hours), DAN (1 μmol/L, 24 hours), DAN (short) (1 μmol/L, 4 hours), or NAC (1 mmol/L, 24 hours). Mitochondrial lipid peroxidation was evaluated using MitoPeDPP (green, middle panels), and mitochondria were double-stained with Mito Tracker Red (red, upper panels). (B) Data summary. Scale bar: 50 μm. Mean ± SEM (n = 33-48 cells from 6 hearts). ∗*P* < 0.05, ∗∗*P* < 0.01, and ∗∗∗*P* < 0.001 by 1-way analysis of variance with Tukey's post hoc test. Abbreviations as in Figure 1.

We investigated the role of RYR2 stabilization in preventing DOX-induced ferroptosis. Isolated cardiomyocytes were treated as shown in Supplemental Figure 7B. Acrolein levels, a marker of lipid peroxidation, increased in a DOX dose–dependent manner in WT cardiomyocytes, but not in RYR2 V3599K-KI cardiomyocytes. After treatment with 1 μmol/L DOX in WT cardiomyocytes, acrolein levels were suppressed in the DOX+DAN (short), DOX+DAN, and DOX+NAC groups (Supplemental Figure 14). Mitochondrial lipid peroxidation was also measured using MitoPeDPP. Similar to the acrolein results, DOX-induced increases in MitoPeDPP levels were significantly reduced in the DOX+DAN (short), DOX+DAN, and DOX+NAC groups (Figure 9).

## Discussion

In this study, we demonstrate that DAN effectively suppresses DOX-induced deterioration of cardiac function and improves prognosis. To clarify this mechanism of action, we further show that genetic stabilization of RYR2 produces similar protective effects, indicating that the reduction of DOX cardiotoxicity is due to RYR2 stabilization rather than an off-target effect of DAN. Specifically, DAN’s anti-DIC effect is mediated by increasing CaM’s binding affinity to RYR2, which prevents Ca^2+^ leakage and subsequently reduces oxidative and ER stress (Central Illustration).

**Central Illustration:**
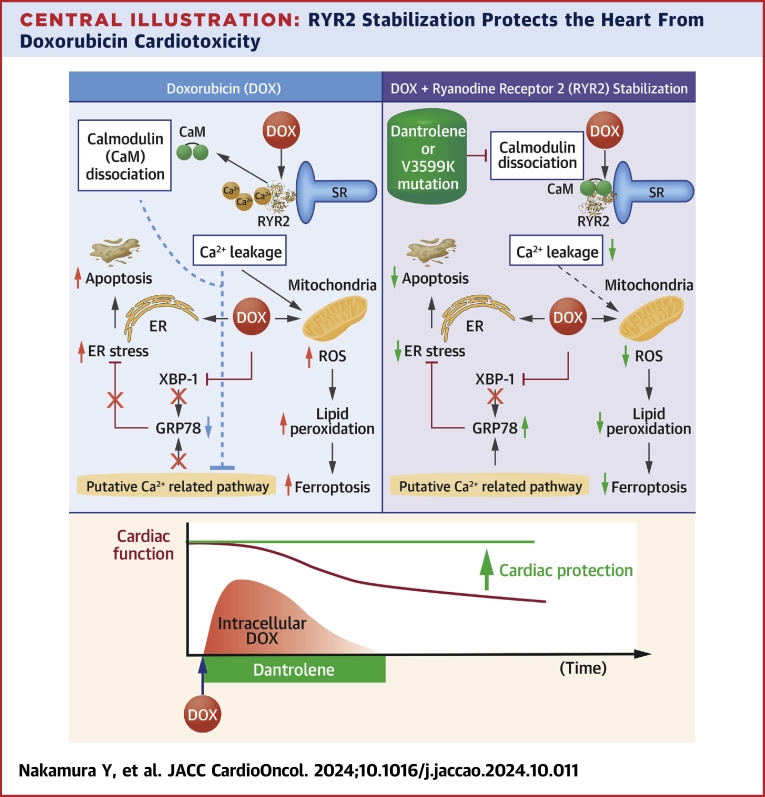
RYR2 Stabilization Protects the Heart From Doxorubicin Cardiotoxicity Apoptosis from endoplasmic reticulum (ER) stress and ferroptosis from reactive oxygen species (ROS), both induced by doxorubicin (DOX), are associated with Ca^2+^ leakage and calmodulin (CaM) dissociation. Stabilizing ryanodine receptor 2 (RYR2) suppresses these effects. Dantrolene (DAN), a RYR2 stabilizer, provides sufficient cardioprotective effects even when administered only during DOX treatment. SR = sarcoplasmic reticulum.

With recent advancements in 3-dimensional analysis of the RYR2 polymer complex at near-atomic resolution,^24^^,^^25^ it has been revealed that the interface between the N-terminal (N: 1-600) and central (C: 2000-2500) domains is located near the binding sites for both CaM (amino acid: 3583-3603) and DAN (amino acid: 601-620) (Supplemental Figure 15). In addition, a recent in-silico molecular docking study identified 2 DOX binding sites within RYR2.^26^ Interestingly, these sites correspond to fragment1-610 and fragment2234-2750, as identified in this study (Supplemental Figure 5B), and are located near the domain–domain interface and CaM binding site (Supplemental Figure 15). This strongly suggests that DOX binding directly to these regions disrupts interdomain interactions within RYR2, leading to CaM dissociation, channel destabilization, and subsequent Ca^2+^ leakage.

Fu et al^27^ reported that DOX decreased expression of spliced XBP-1, leading to a subsequent decrease in GRP78 expression, which induced apoptosis due to excessive ER stress. By contrast, overexpression of spliced XBP-1 increases GRP78 expression, mitigating DOX-induced cardiomyocyte death.^27^ In this study, both the RYR2 V3599K mutation and DAN restored GRP78 expression after DOX exposure, without altering XBP-1 levels (Figure 8, Supplemental Figures 12 and 13). This suggests that RYR2 stabilization is closely linked to GRP78 expression, independent of XBP-1 regulation. However, the precise pathway remains undetermined and is an important topic for future research.

Ferroptosis, caused by glutathione peroxidase 4 depletion and elevated ROS, plays a key role in DOX-induced cell death.^28^ Intracellular Ca^2+^ has been implicated in promoting ferroptotic cell death, as shown in a neuronal cell line.^29^, ^30^, ^31^, ^32^ CoCl_2_, a general inhibitor of Ca^2+^ channels, protects cells from glutamate-induced oxytosis and ferroptosis induced by erastin-1 or RSL3.^29^^,^^32^ In addition, ruthenium red, which inhibits mitochondrial Ca^2+^ uptake, prevents mitochondrial ROS production and cell death from glutamate toxicity.^29^ These results suggest that Ca^2+^ leakage through RYR2 is taken up by mitochondria, significantly contributing to ROS production and subsequent ferroptosis.

DOX also affects other cellular compartments, such as mitochondria and the nucleus, where direct damage occurs regardless of DAN presence (Supplemental Figures 16 and 17). However, in clinical settings, Ca^2+^ leakage from RYR2 appears to be a primary mechanism of DIC shortly after DOX administration. This is supported by findings that short-term DAN administration or the V3599K RYR2 mutation provided sufficient cardioprotection despite DOX-induced nuclear and mitochondrial damage (Figure 6C, Supplemental Figure 6). The cardioprotective effects of short-term DAN use suggest a highly feasible clinical strategy. Furthermore, because DAN is already used safely in clinical settings to treat malignant hyperthermia (a disease caused by RYR1 point mutations), these results hold promise for its potential repositioning in the near future.

### Study limitations

Because we did not use a cancer model, we could not determine whether DAN interferes with the chemotherapeutic effects of DOX. However, a previous study in a breast cancer model showed that DAN attenuated DOX cardiotoxicity without diminishing its antitumor efficacy.^16^ Additionally, sex differences in susceptibility to DIC have been reported.^33^^,^^34^ Although no sex differences were observed in our echocardiographic data (Supplemental Figure 11), we cannot rule out the possibility of sex-related differences in measures beyond cardiac function.

## Conclusions

This study demonstrates that enhancing CaM binding affinity to RYR2 and suppressing Ca2+ leakage effectively prevent DIC and improves prognosis. Notably, short-term DAN administration together with DOX proved sufficient for cardioprotection (Central Illustration). This combined therapy of DOX and DAN, leveraging DAN’s established clinical safety, offers a promising and innovative approach for preventing DIC in clinical settings.

Perspectives **COMPETENCY IN MEDICAL KNOWLEDGE:** Ca^2+^ leakage or CaM dissociation are important triggers of doxorubicin-induced cardiotoxicity, and enhancing CaM binding affinity to RYR2 is an effective strategy for preventing doxorubicin-induced cardiotoxicity.

**TRANSLATIONAL OUTLOOK:** Further research is needed to evaluate the effectiveness of short-term dantrolene use for cardioprotection in patients receiving doxorubicin chemotherapy.

## Funding Support and Author Disclosures

This work is supported by UBE Industries Foundation, the Shinnihon Foundation of Advanced Medical Treatment Research, Grant-in-Aid for Scientific Research B (23H02906) and Grant-in-Aid for Early-Career Scientists (24K19034). This work was the result of using research equipment shared in the MEXT Project for promoting public utilization of advanced research infrastructure (Program for supporting construction of core facilities) Grant Number JPMXS0440400023. The authors have reported that they have no relationships relevant to the contents of this paper to disclose.
