# Supplementary material for: Concomitant Administration of Dantrolene is Sufficient to Protect Against Doxorubicin-Induced Cardiomyopathy
Source: JACC CardioOncol. 2024 Dec 10;7(1):38–52. doi: 10.1016/j.jaccao.2024.10.011 (PMC11782101; doi:10.1016/j.jaccao.2024.10.011)
Supplement: Supplemental Material [file mmc1.docx]

**Supplemental Appendix**

**for**

**Concomitant administration of dantrolene is sufficient to protect against doxorubicin-induced cardiomyopathy**

Yoshihide Nakamura^a^*, Takeshi Yamamoto^b^, Shigeki Kobayashi^a^, Takeshi Suetomi^c^, Hitoshi Uchinoumi^c^, Tetsuro Oda^c^, Motoaki Sano^c^, Masafumi Yano^c^

^a^Department of Therapeutic Science for Heart Failure in the Elderly, Yamaguchi University Graduate School of Medicine, Ube, Japan

^b^Faculty of Health Sciences, Yamaguchi University Graduate School of Medicine, Ube, Japan

^c^Department of Medicine and Clinical Science, Division of Cardiology, Yamaguchi University Graduate School of Medicine, Ube, Japan

**Supplemental Methods – pages 2 - 10**

**Supplemental Figures 1-17 – pages 11 - 32**

**Data Availability – page 32**

**Supplemental References – pages 33**

**Supplemental Methods**

*Animals.* Male and female C57BL/6 mice were used in the present study. WT C57BL/6 mice were obtained from Japan SLC, Inc. The RYR2^V3599K-KI^ mice were purchased from UNITECH Co. Ltd (Bangkok, Thailand). DOX-induced cardiomyopathy mouse models were generated as previously reported, with some modifications.^1, 2^ Briefly, DOX (6 mg/kg body weight) was administered intraperitoneally to 8-week-old mice on days 0, 7, 14, and 21. The mice were randomly assigned to each experiment, and the order of the groups in each experiment was decided randomly. All strains were maintained on a C57BL/6 background. The measurement and analysis of the acquired data were performed by investigators who were blinded to the genetic information of the mice.

This study conformed to the Guide for the Care and Use of Laboratory Animals published by the National Academies Press. The care of the animals and the protocols used were in accordance with the guidelines of the Animal Ethics Committee of Yamaguchi University Graduate School of Medicine.

*Dantrolene administration.* *In vivo* experiments, using a feeding apparatus (Roden CAFÉ; Oriental Yeast Co., Ltd., Tokyo, Japan), dantrolene-treated mice were fed 100 mg/kg/day dantrolene (Fuji Film Wako Chemicals, Tokyo, Japan). The oral dose of dantrolene for chronic administration was determined as the dose by which inducible ventricular tachycardia was almost completely inhibited in CPVT-type RYR2R2474S+/− KI mice.^3, 4^ Dantrolene administration was initiated 1 week before DOX administration. In DOX+DAN (short) group, dantrolene treatment ended at 1 week after the last DOX administration. In vitro experiments, 1 µM dantrolene was added to cardiomyocytes 30 min before DOX administration. In DOX+DAN (short) group, isolated cardiomyocytes were treated with DOX+dantrolene for 4 h, after which medium without DOX+dantrolene was substituted. Cardiomyocytes were evaluated 24 h after the start of the experiments (Supplemental Figure 7).

*Reagents.* DOX (D41933) was purchased from the Tokyo Chemical Industry (Tokyo, Japan). DOX-SMCC (HY-116063) was purchased from MedChemExpress (Monmouth Junction, NJ, USA). Dantrolene (D9175) was purchased from Sigma-Aldrich (St Louis, MO, USA). NAC (015-05132) was purchased from FUJIFILM Wako Pure Chemical Corp. (Osaka, Japan).

*Echocardiogram.* Cardiac function was analyzed using an F37 ultrasound machine (Hitachi Medical, Chiyoda, Japan) equipped with a 7.5-MHz probe (Hitachi; UST-5413). Mice were initially anesthetized with 4–5% isoflurane (mixed with oxygen) and anesthesia was maintained with 1–2% isoflurane during echocardiography. Echocardiographic findings were obtained in parasternal long-axis view at the level of the papillary muscles. The Devereux formula was used to calculate left ventricular (LV) mass.^5^

*Isolation of cardiomyocytes.* Cardiomyocytes were isolated from mouse hearts as described previously.^3^ Briefly, the mice were anesthetized with pentobarbital sodium (70 mg/kg body weight, intraperitoneally), intubated, and ventilated with ambient air. An incision was made in the chest and the heart was quickly removed and retrogradely perfused with collagenase-free buffer (Thermo Fisher Scientific, Waltham, MA, USA; DMEM, 10313) via the aorta under constant flow. The left ventricular myocardium was then minced with scissors into a fresh collagenase-containing buffer, and the rod-shaped adult mouse cardiomyocytes were prepared by retrograde perfusion of the hearts with 95% O_2_ and 5% CO_2_-bubbled minimal essential medium (MilliporeSigma, Burlington, MA, USA) supplemented with 50 μM [Ca^2+^], 0.5 mg/ml collagenase B (Roche, Basel, Switzerland), 0.5 mg/ml collagenase D (Roche), and 0.02 mg/ml protease type XIV (MilliporeSigma, P-5147). The Ca^2+^ concentration was then gradually increased to a final concentration of 1 mM by changing the incubation medium (50, 100, 300, and 600 μM, and then 1 mM). Isolated mouse cardiomyocytes were transferred to laminin-coated glass culture dishes and incubated for a few hours at 37°C in a 5% CO_2_ and 95% O_2_ atmosphere.

*Monitoring of Ca^2+^ dynamics.* Intracellular Ca^2+^ measurements were performed as previously described using a laser-scanning confocal microscope (LSM-510, Carl Zeiss, Oberkochen, Germany) equipped with an argon ion laser coupled to an inverted microscope (Axiovert 100, Carl Zeiss) and a Zeiss ×40 oil-immersion Plan-Neofluor objective (numerical aperture, 1.3; excitation at 488 nm; emission at > 505 nm).^3^ Intact cardiomyocytes were incubated with 20 μM Fluo-4 AM (DOJINDO laboratories, Kumamoto, Japan) for 20 min at room temperature and washed twice with Tyrode’s solution (made in-house; 140 mM NaCl, 1 mM MgCl2, 10 mM glucose, 6 mM KCl, 5 mM HEPES pH7.4, 2 mM CaCl_2_). Line-scan mode was used to measure Ca^2+^ sparks and Ca^2+^ transients, in which a single cardiomyocyte was scanned repeatedly along a line parallel to the longitudinal axis, avoiding the nuclei. Ca^2+^ transients were measured at 2-Hz pacing using a field electric stimulator (IonOptix, Westwood, MA, USA).

Ca^2+^ events under fixed intracellular Ca^2+^ conditions were measured in saponin-permeabilized cardiomyocytes. Ventricular myocytes were superfused with relaxing solution (made in-house; EGTA 0.1 mmol/L, ATP 5 mmol/L, HEPES 10 mmol/L, potassium aspartate 150 mmol/L, MgCl_2_ 0.25 mmol/L, and reduced-glutathione 10 mmol/L). The sarcolemma was permeabilized with saponin (50 µg/mL) for 30 s. After permeabilization, myocytes were placed in a solution containing EGTA 0.5 mmol/L, HEPES 10 mmol/L, K-aspartate 120 mmol/L, ATP 5 mmol/L, free MgCl_2_ 1 mmol/L, reduced-glutathione 10 mmol/L, free [Ca_2+_] 40 nmol/L (calculated using MaxChelator (http://www.stanford.edu/~cpatton/webmaxcS.htm)), creatine phosphokinase 5 U/ml, phosphocreatine 10 mmol/L, dextran (Mr: 40,000) 4%; Fluo3 (DOJINDO, Kumamoto, Japan) 20 µM, pH 7.2. Ca^2+^ spark images were obtained before and after the addition of cAMP (0.1–1 µmol/L) or thapsigargin (0.3 µmol/L). To assess the SR Ca^2+^-content, caffeine (10 mM) was rapidly perfused to discharge SR-loaded Ca^2+^.

*Monitoring of intracellular reactive oxygen species.* A fluorescent probe, 2′,7′-dichlorofluorescin diacetate (DCFH-DA, Thermo Fisher Scientific), was used to assess intracellular ROS formation. Intact cardiomyocytes were incubated with 1 μM DCFH-DA for 20 min at 37°C and were washed twice with Tyrode’s solution. At the end of the observation, H_2_O_2_ was added to evaluate the max value. Fluorescent images were obtained using a fluorescent digital microscope (BZ9000, Keyence, Japan; Plan 20 × Objective, Nikon, Japan).

*Immunocytochemistry analysis.* Antibodies against RYR2 (C3-33 1/250, Sigma-Aldrich), CaM (EP799Y 1/250, Abcam, Cambridge, UK), GRP78 (PA5-19503 1/600, Thermo Fisher Scientific), XBP-1 (EPR4086 1/1000, Abcam) and Alexa Fluor® 488 mouse anti-H2AX (pS139) (#560445 1/200, BD) were used for immunocytochemistry. Isolated cardiomyocytes were fixed with 4% paraformaldehyde (WAKO, 163-20145) in phosphate-buffered saline (PBS) for 5 min, washed three times with PBS, and permeabilized in 0.5% Triton X-100 (Millipore Sigma) and 1% bovine serum albumin (BSA, Nacalai Tesque, Kyoto, Japan) for 20 min. Then, the cardiomyocytes were incubated overnight at 4°C with the primary antibodies in 1% BSA and 0.5% Triton X-100, followed by labeling with an Alexa Fluor 488-conjugated secondary antibody (1/300, for CaM, GRP78 and XBP-1), Alexa Fluor 633-conjugated secondary antibody (1/300, for RYR2). The cardiomyocytes were washed three times with PBS.

*Assessment of sDOX fluorescence.* The binding of DOX to dog SR or recombinant RYR2 fragments was evaluated using the DOX-SMCC (sDOX). sDOX is an agent-linker conjugate for antibody-drug conjugate (ADC). RYR2 fragments are the peptides corresponding to various regions of RYR2 (1-610, 740-1260, 1245-1768, 1741-2270, 2234-2750). Detailed methods are described previously.^6^ sDOX (1 μM) was mixed with target protein for 1 min. The reaction was stopped by the adding of lysin (5mM). Sample were run on a gel, and sDOX fluorescence in gel was measured on a blue LED transilluminator (LEDB-SBOXHP).

*Assessment of endogenous RYR2-bound CaM.* The sarcomere-related periodical increase in the Alexa Fluor 633 and Alexa Fluor 488 fluorescence intensity from baseline was integrated with the longitudinally selected distance (ca. 25 μm), and then the value was divided by the distance. The mean value of one sarcomere-related increase in fluorescence intensity was calculated as the arbitrary amount of RYR and RYR-bound CaM. Immunofluorescence signal of CaM was divided by that of RYR2, normalized to WT control and expressed as a ratio. Secondary antibody labeling alone showed no detectable fluorescence.

*Measurements of mitochondrial lipid peroxidation and superoxide.* Lipid peroxides in the mitochondrial inner membrane was assessed using MitoPeDPP (DOJINDO Laboratories, Kumamoto, Japan), a fluorescent probe that specifically reacts with lipid peroxides in the mitochondrial inner membrane. Mitochondrial superoxide was assessed using MitoSOX Green (Thermo Fisher Scientific, Waltham, USA). The mitochondria were counterstained with MitoTracker Red (Thermo Fisher Scientific, Waltham, USA). Cultured cells were incubated in the presence of 0.5 μM MitoPeDPP or 0.5 μM MitoSOX and 0.5 μM MitoTracker solution for 30 min at 37°C. Following washing with Tyrode’s solution, mitochondrial lipid peroxidation and superoxide were measured fluorometrically (excitation, 488 nm; emission: 505–545 nm, excitation, 633 nm; emission, > 650 nm) using a confocal microscope (LSM-510, Carl Zeiss).

*Histology.* Mouse hearts were fixed in 10% formalin 4 weeks after the completion of DOX administration. A complete, fully circumferential section at the level of the two left ventricular papillary muscles was selected for morphometric analysis. The extent of fibrosis was analyzed using Picro Sirius Red staining (SCY, PSR-1). Red-stained areas were quantified using ImageJ software. TUNEL staining was performed to detect intracellular DNA fragments using the In Situ Cell Death Detection Kit, Fluorescein (Roche). Cell membranes were stained with wheat germ agglutinin Alexa Fluor 488 conjugate (VEC, FL-1021) for cross sectional area quantification.

*Immunoblot analysis.* Homogenate samples prepared from hearts or cultured cardiomyocytes were obtained. The hearts were removed from mice and washed with ice-cold PBS, and the cultured cells were harvested using a cell scraper. Both were homogenized in lysis buffer containing protease inhibitor cocktail followed by centrifugation at 4000×g for 10 min at 4 ℃. The supernatant was collected followed by centrifugation at 20000×g for 15 min at 4 ℃. The pellet was resuspended, adjusted for concentration, and denatured in SDSPAGE sample buffer. SDS-PAGE, blotting, and antibody detections were performed using the following primary antibodies: RYR2 (Millipore Sigma, NR07100UG), GRP78 (Thermo Fisher Scientific, PA5-19503), P53 (Cell Signaling Technology, Danvers, MA, USA; 9282), Caspase-12 (Sigma-Aldrich, C7611), acrolein (JalCA, MAR-020n), SirT3 (Cell Signaling Technology, #5490S) and glyceraldehyde-3-phosphate dehydrogenase (Millipore Sigma).

*Statistical analysis.* Two-tailed Student’s t-tests were used for statistical comparisons of data obtained under two different conditions, whereas one-way analysis of variance (ANOVA) followed by a post-hoc Tukey’s test or Dunnett’s test was used for statistical comparisons of more than two groups. A repeated measures ANOVA was used to control for the correlation within mouse. Normal distribution of the data was determined by the Shapiro-Wilk test, which was performed using JMP pro 16 software (JMP Statistical Discovery LLC, Cary, North Caroline, USA). All other statistical analysis and plotting were performed using GraphPad Prism 5 software (GraphPad Software, LLC, Boston, MA, USA). All data are expressed as mean ± standard error of the mean. Statistical significance was set at p < 0.05. To investigate survival, Kaplan-Meier plots were generated for each group and a log-rank test was performed.

*Study approval.* All experiments were approved by the Animal Ethics Committee of the Yamaguchi University Graduate School of Medicine.


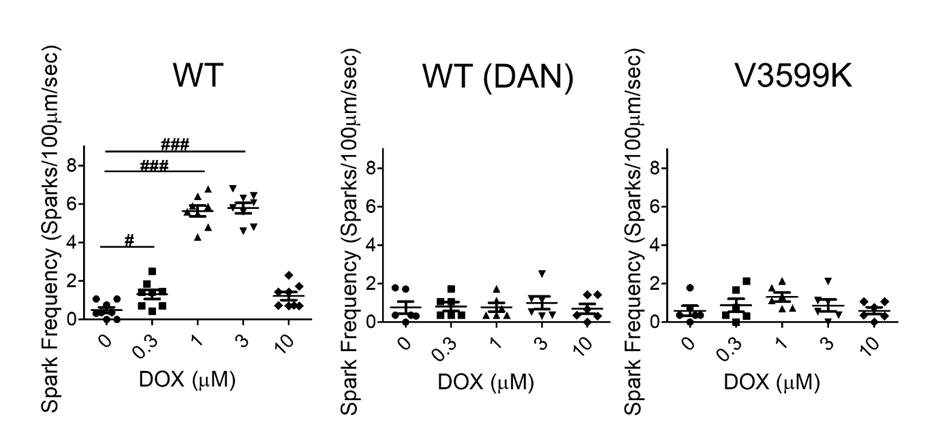


**Supplemental Figure 1. Ca^2+^ spark up to 5 min after DOX administration** Changes in Ca^2+^ spark depending on concentration (0, 0.3, 1, 3, 10 μM) up to 5 min after DOX administration in WT, WT treated with 1 μM dantrolene (WT [DAN]) and V3599K-KI cardiomyocytes. Values for individual mice are plotted with mean ± standard error of the mean. #P < 0.05, ###P < 0.001 (repeated measures ANOVA with Dunnett's post-hoc test); n = 30–42 cells from 6–8 hearts.

DAN = dantrolene; DOX = doxorubicin; KI = knock-in; ROS = reactive oxygen species; WT = wild-type.


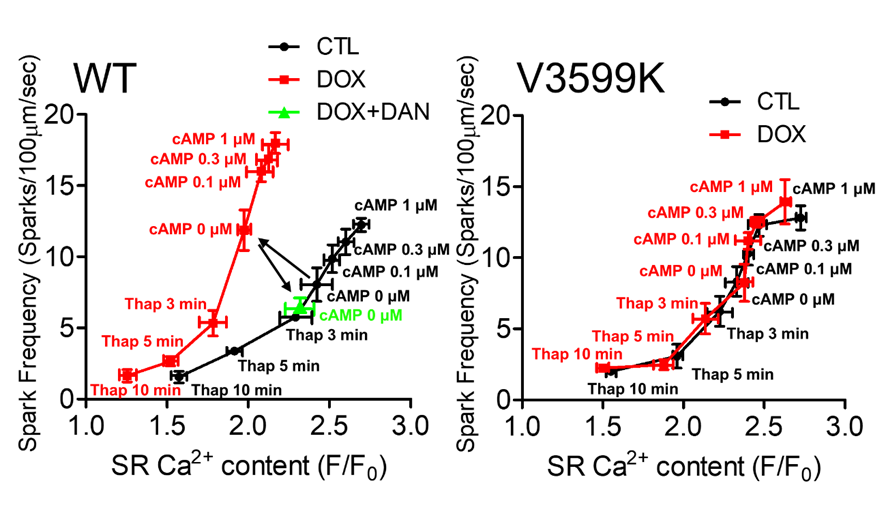


**Supplemental Figure 2. Relationship between Ca^2+^ spark frequency and SR Ca^2+^-content** Relationship between Ca^2+^ spark frequency (SpF) and SR Ca^2+^-content with or without DOX (1 μM) in saponin-permeabilized WT and V3599K-KI cardiomyocytes. The arrow indicates the shift of the data by the addition of DOX (1 μM) in the absence or presence of dantrolene (WT [n = 4 hearts]: [(-)DOX: 12–20 cells for SpF and 10 cells for SR Ca^2+^-content; (+)DOX: 14–24 cells for SpF and 9 cells for SR Ca^2+^-content; (+)DOX, (+)dantrolene: 10 cells for SpF and five cells for SR Ca^2+^-content], KI [n = 4 hearts]: [(-)DOX: 14–20 cells for SpF and 9 cells for SR Ca^2+^-content; (+)DOX: 13–21 cells for SpF and 5 cells for SR Ca^2+^-content])

CTL = control; DAN = dantrolene; DOX = doxorubicin; KI = knock-in; SpF = Ca^2+^ spark frequency; SR = sarcoplasmic reticulum; WT = wild-type.


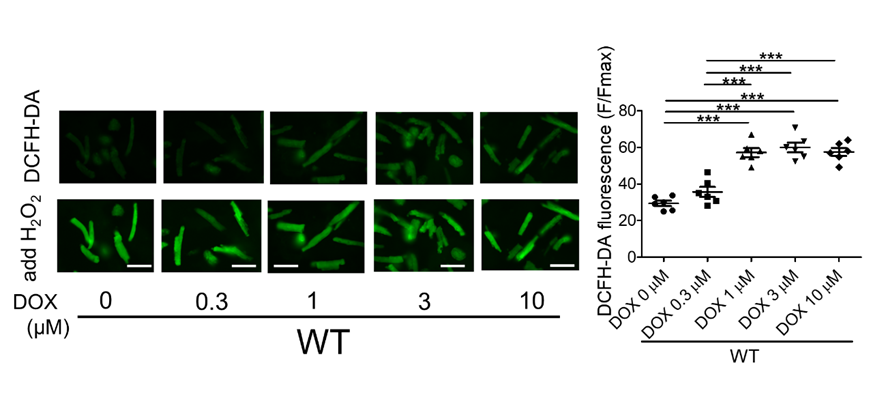


**Supplemental Figure 3. ROS levels after 24h cultured WT cardiomyocytes** Representative images of DCFH-DA fluorescence depending on DOX concentration (0, 0.3, 1, 3, 10 μM) after 24h. cultured WT cardiomyocytes (left), and summarized data (right). Scale bar: 100 μm. Values for individual mice are plotted with mean ± standard error of the mean. ***P < 0.001 (repeated measures ANOVA with Tukey's post-hoc test); n = 98–119 cells from 6 hearts.

DOX = doxorubicin; ROS = reactive oxygen species; WT = wild-type.


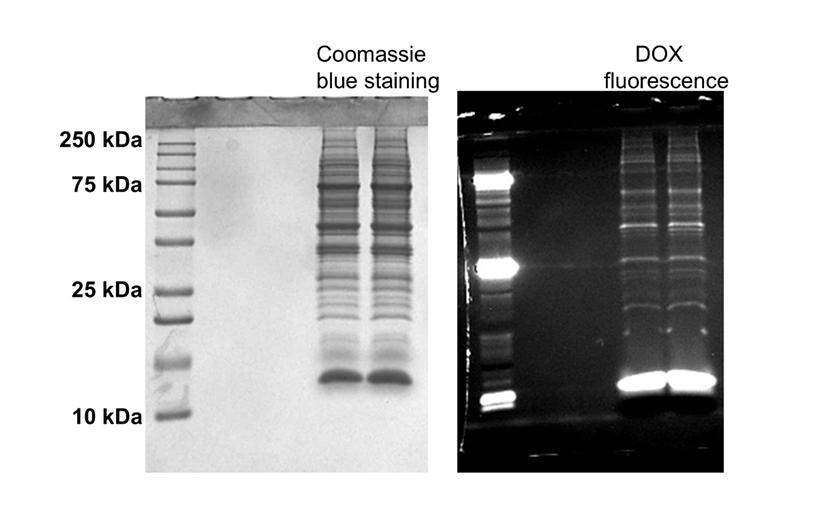


**Supplemental Figure 4. DOX binding to crude heart homogenates** Representative images of sDOX fluorescence mixed with crude heart homogenates (right) and Coomassie brilliant blue staining of the same gel (left).

sDOX = DOX-SMCC.


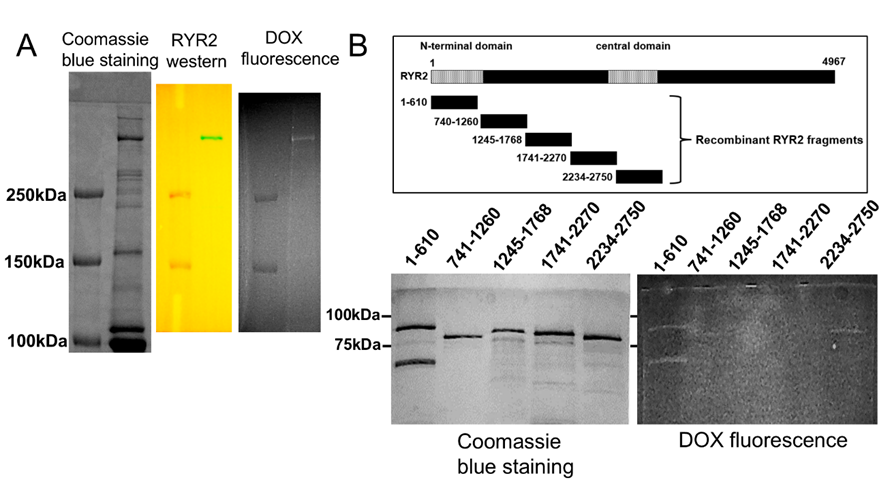


**Supplemental Figure 5. Direct binding of DOX to RYR2** (A) Representative images of Coomassie brilliant blue staining (left), western blot of RYR2 (middle) of SR vesicle and sDOX fluorescence mixed with SR vesicle (right). Bands were observed at various heights in the 6% SDS gel stained with Coomassie brilliant blue, but the fluorescence was localized at the same height as that of the RYR2 band in corresponding western blots. (B) Representative images of sDOX fluorescence mixed with recombinant RYR2 fragments. The fluorescences were observed specifically in fragment1-610 and fragment2234-2750.

sDOX = DOX-SMCC; SR = sarcoplasmic reticulum.


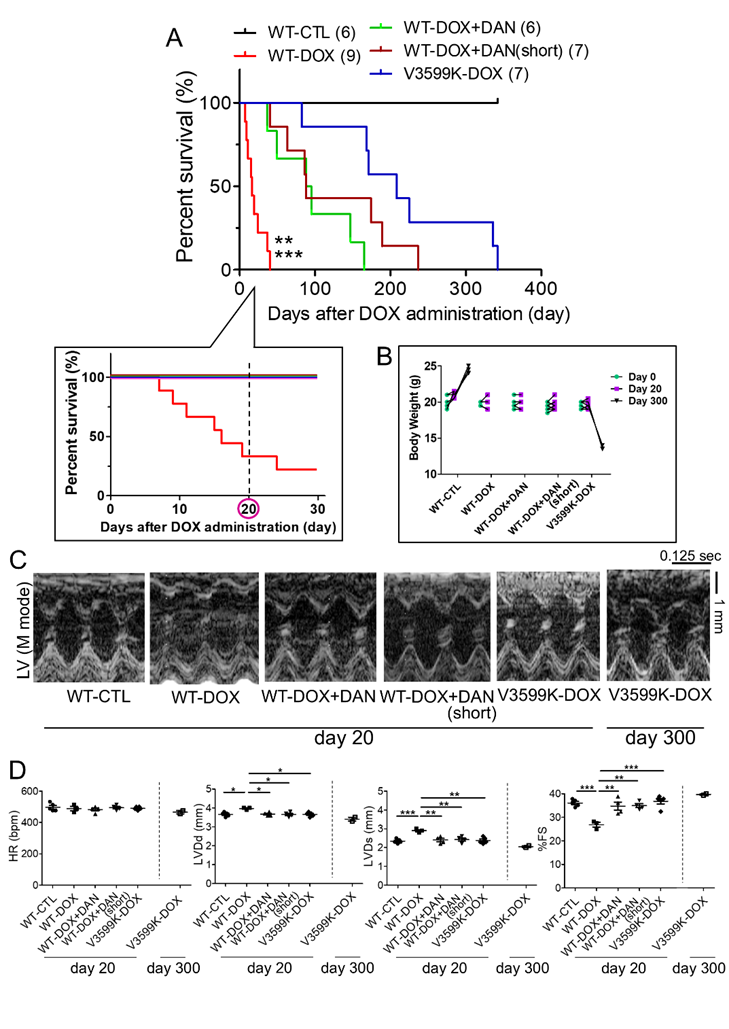


**Supplemental Figure 6. Kaplan-Meier survival analysis after high-dose DOX administration**

(A) A single dose of DOX (24 mg/kg) was injected into the WT and V3599K-KI mice. Dantrolene was administered continuously (WT-DOX+DAN) and discontinued after 1 week of DOX administration (WT-DOX+DAN [short]). Values in parentheses indicate the number of mice. **P < 0.01 versus WT-DOX+DAN and WT-DOX+DAN (short), ***P < 0.001 versus WT and V3599K-DOX (log-rank test). (B) Weight changes from start to day20 (all group) and day 300 (V3599K-DOX) (middle right) (n=2-7). (C) Representative echocardiographic images at day 20 (all groups) and day 300 (V3599K-DOX). (D) summarized data. (n=2-5, mean ± SEM, *P < 0.05, **P < 0.01, ***P < 0.001, ANOVA with Tukey's post-hoc test).

KI = knock-in; DAN = dantrolene; DOX = doxorubicin; LVDd/Ds = left ventricular end-diastolic diameter/end-systolic diameter; %FS = percentage fractional shortening; WT = wild-type.


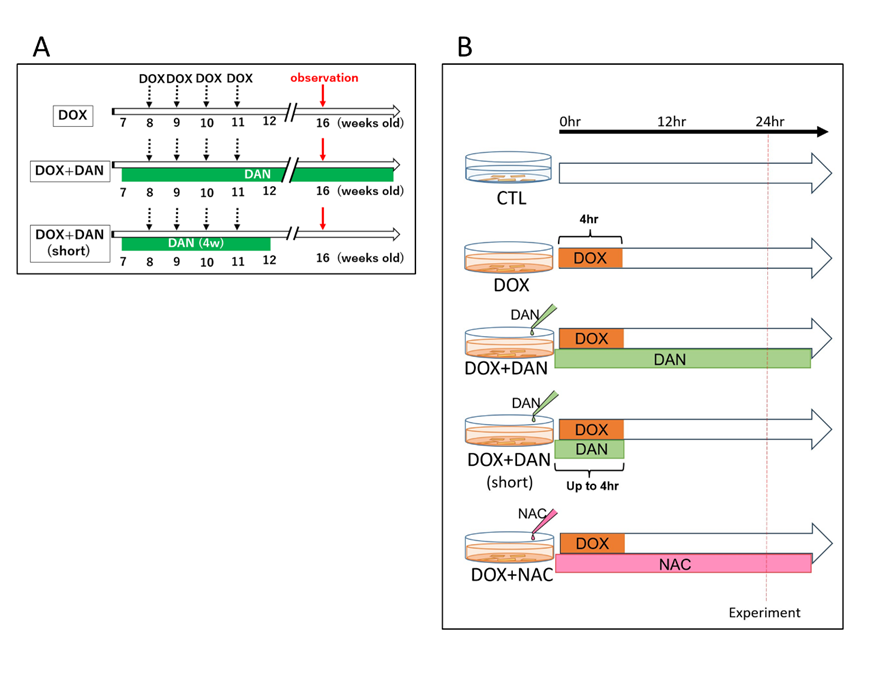


**Supplemental Figure 7. Protocol for chronic (*in vivo*) and acute (*in vitro*) experiments** (A) Chronic experimental protocol for DOX administration and dantrolene treatment *in vivo*. All mice were evaluated at 16 weeks of age. (B) Acute experimental protocol for DOX, DAN and NAC administration *in vitro*. Isolated cardiomyocytes were cultured in the presence of DOX (1 μM, 4 h), DAN (1 μM, 24 h), DAN (short) (1 μM, 4 h), or NAC (1 mM, 24 h), after which medium without DOX was substituted, and cells were evaluated at 24 h.

DAN = dantrolene; DOX = doxorubicin; NAC = N-acetyl cysteine.


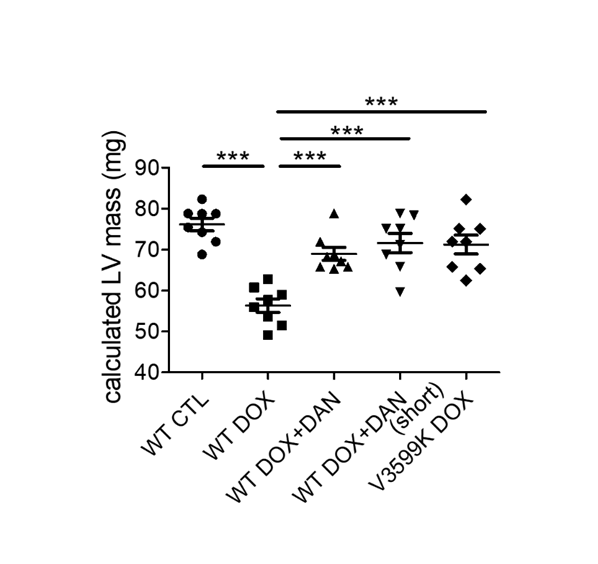


**Supplemental Figure 8. Evaluation of LV mass** Calculated LV mass of chronic experimental mice on echocardiography. N=8 mice, values for individual mice are plotted with mean ± SEM. ***P < 0.001 (ANOVA with Tukey's post-hoc test).

DAN = dantrolene; DOX = doxorubicin; LV=left ventricle; WT = wild-type


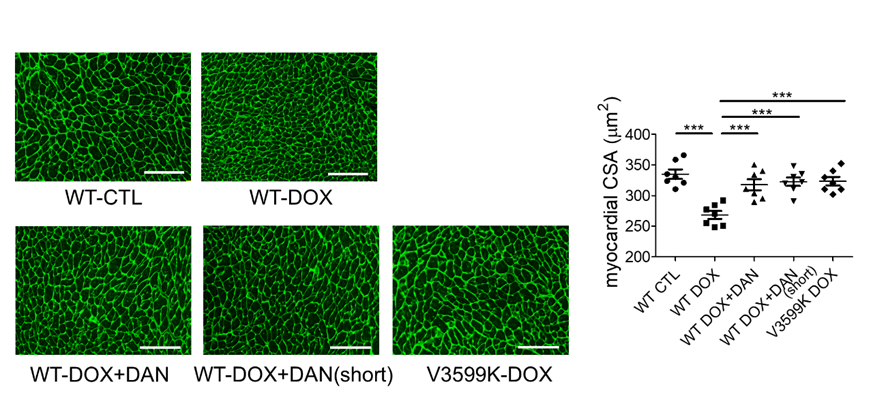


**Supplemental Figure 9. Cardiomyocyte cross-sectional area** Representative images of heart sections of chronic experimental mice stained with wheat germ agglutinin (left), and summarized data (right). Scale bar: 100 μm. Values for individual mice are plotted with mean ± standard error of the mean. ***P < 0.001 (ANOVA with Tukey's post-hoc test); n = 700-900 cross-sections from 7 hearts.

DAN = dantrolene; DOX = doxorubicin; WT = wild-type; CSA = cross-sectional area.


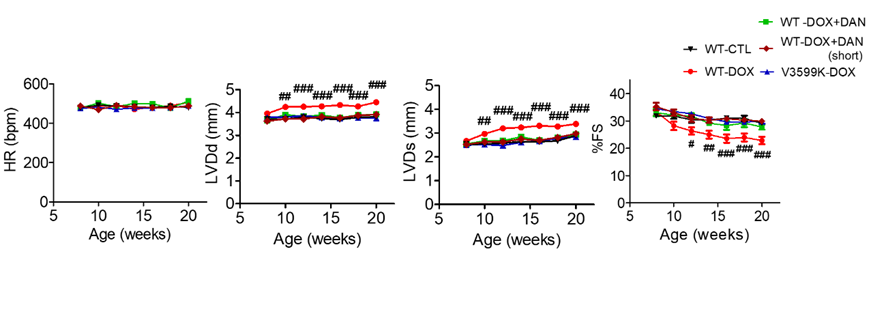


**Supplemental Figure 10. Changes in cardiac function over time in chronic model mice** Progress of echocardiographic findings every 2 W after starting DOX administration (n = 9-11). #P < 0.05, ##P < 0.01, ###P < 0.001 versus WT CTL at the same age (ANOVA with Dunnett’s post-hoc test). DAN = dantrolene; DOX = doxorubicin; WT = wild-type.


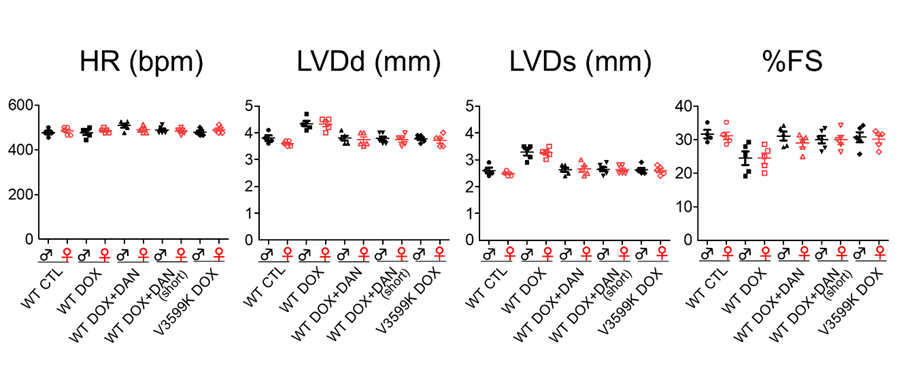


**Supplemental Figure 11. Sex differences in echocardiographic findings** Echocardiographic findings in male and female mice between groups at 16 weeks old in DOX chronic administration model. There were no significant differences between the sexes in each group. DAN = dantrolene; DOX = doxorubicin; WT = wild-type.


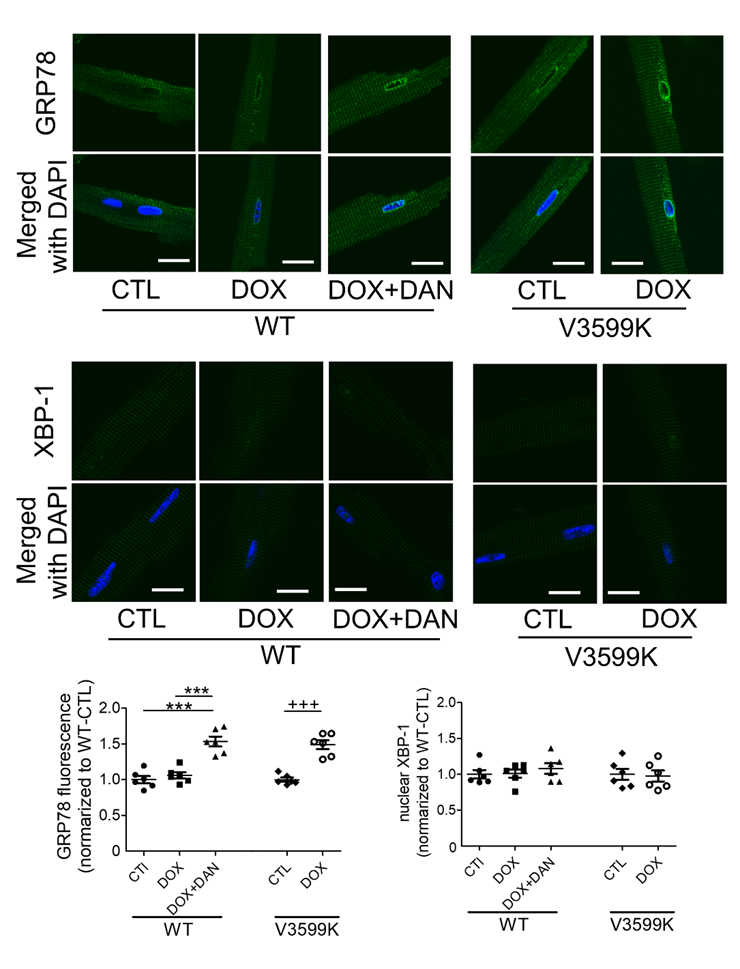


**Supplemental Figure 12. Evaluation of GRP78 and XBP-1** Representative images of GRP78 and XBP-1 evaluated by immunostaining cardiomyocytes isolated from mice

under control conditions (CTL), at 5 days after DOX (6 mg/kg body weight) administration (DOX), and 5 days after DOX administration with oral DAN administration (DOX+DAN) (Top), and summarized data (Bottom). Scale bar: 25 μm. N = 52–67 cells from 6 hearts. Values for individual mice are plotted with mean ± SEM. ***P < 0.001 (ANOVA with Tukey's post-hoc test). +++P < 0.05 (unpaired Student’s t test).

DAN = dantrolene; DOX = doxorubicin; WT = wild-type.


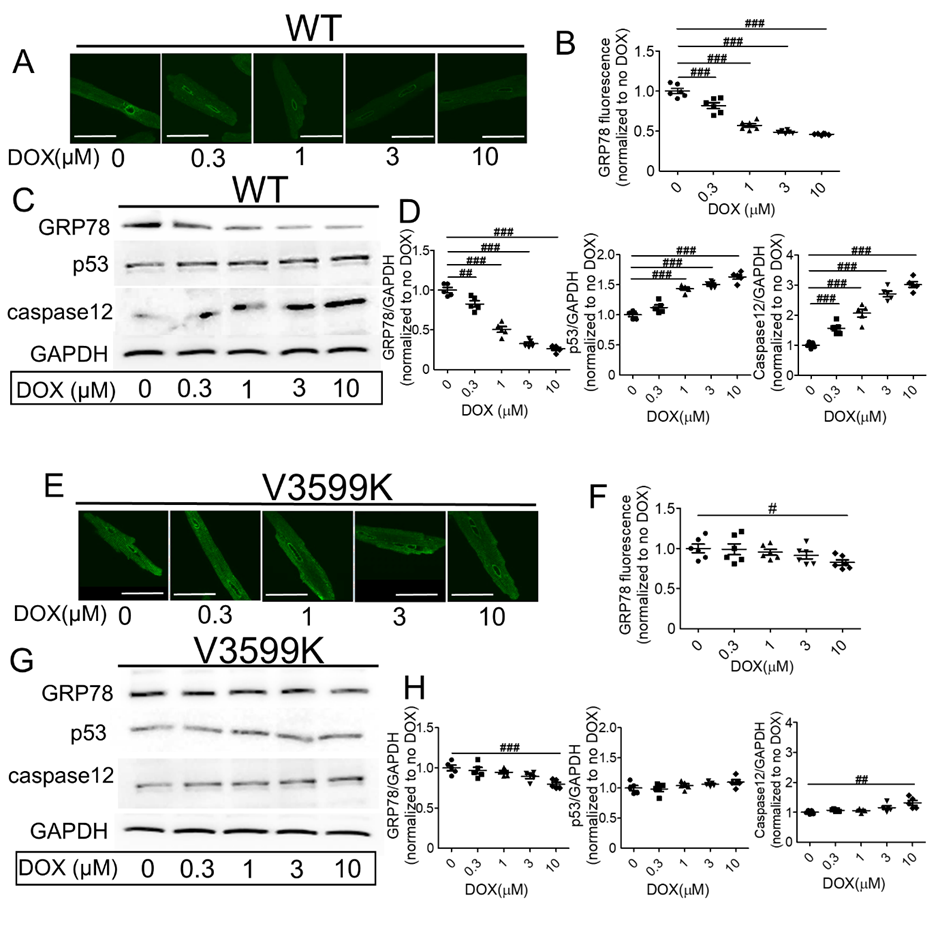


**Supplemental Figure 13. Evaluation of ER stress** Changes in ER stress markers depending on DOX concentration in 24h cultured WT cardiomyocytes. (A) Representative immunostaining images of GRP78 (left), and (B) summarized data. N = 33-38 cells from 6 hearts. (C) Western blots of GRP78, P53, caspase-12, and GAPDH in lysates from 24h cultured cardiomyocytes, and (D) summarized data. N = 5 hearts. Changes in ER stress markers depending on DOX concentration in 24h cultured V3599K-KI cardiomyocytes. (E) Representative immunostaining images of GRP78, and (F) summarized data. N = 35-42 cells from 6 hearts. (G) Western blots of GRP78, P53, caspase-12, and GAPDH in lysates from 24h cultured cardiomyocytes, and (H) summarized data. N = 5 hearts.

Scale bar: 50 μm. Values for individual mice are plotted with mean ± SEM. #P < 0.05, ##P < 0.01, ###P <0 .001 (repeated measures ANOVA with Dunnett's post-hoc test).

ER = endoplasmic reticulum; KI = knock-in; DAN = dantrolene; DOX = doxorubicin; NAC = N-acetyl cysteine; WT = wild-type.


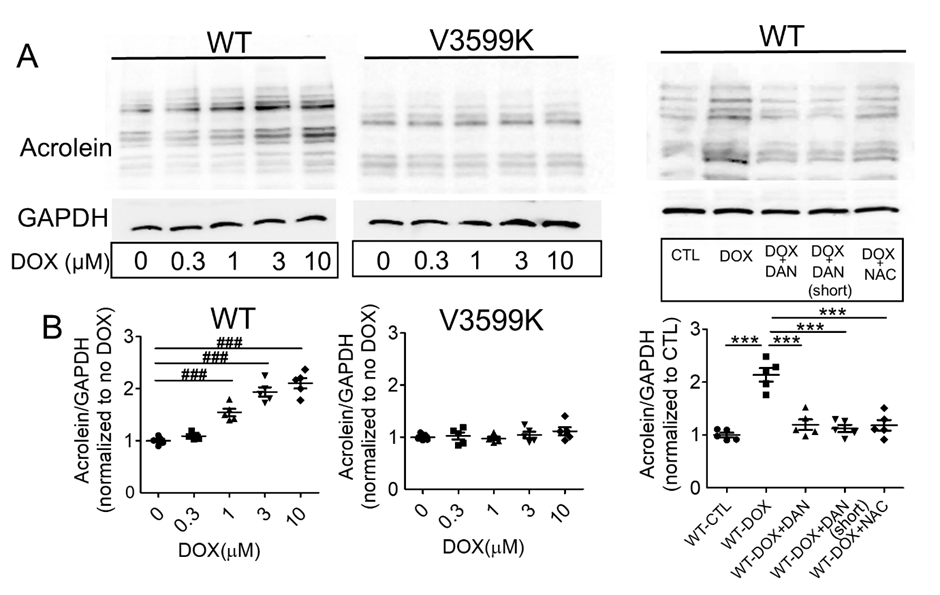


**Supplemental Figure 14. Evaluation of ferroptosis** (A) Representative images of western blots of acrolein and GAPDH in lysates from 24h cultured cardiomyocytes, and (B) summarized data. (n = 5 hearts, mean ± SEM, ###P < 0.001: repeated measures ANOVA with Dunnett's post-hoc test, ***P < 0.001: repeated measures ANOVA with Tukey's post-hoc test).

DAN = dantrolene; DOX = doxorubicin; NAC = N-acetyl cysteine; WT = wild-type.


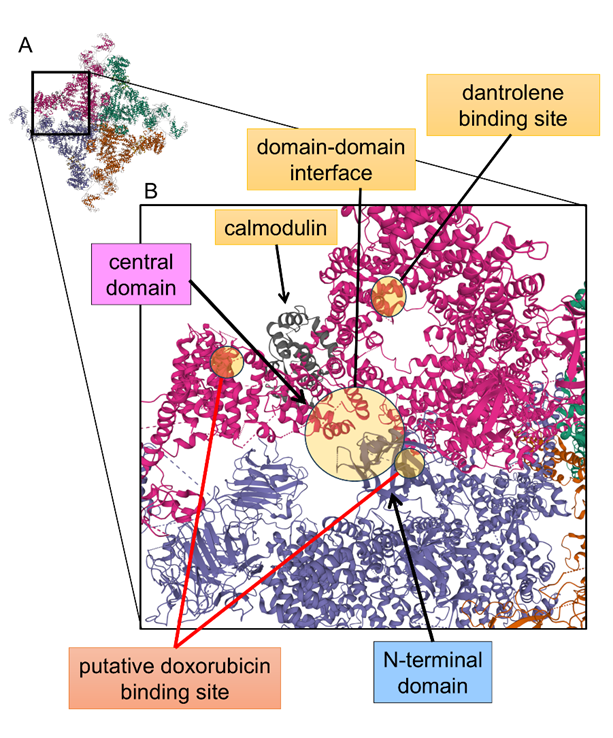


**Supplemental Figure 15. 3-dimensional structure of RYR2** (A) Plan view of the RYR2 tetramer structure. (B) Enlarged view near the N-terminal and central domain interface. N-terminal (amino acid 1-220; blue) and central (amino acid 2250-2500; pink) domain interact with each other. The CaM binds near this interface and DAN (amino acid 601-620) binds near the CaM binding domain (amino acid 3583-3603). The DOX binding sites predicted from recombinant RYR2 fragments (fragment1-610 and fragment2234-2750) are located near the CaM binding site and domain-domain interface.

CaM = calmodulin; DAN = dantrolene; DOX = doxorubicin.


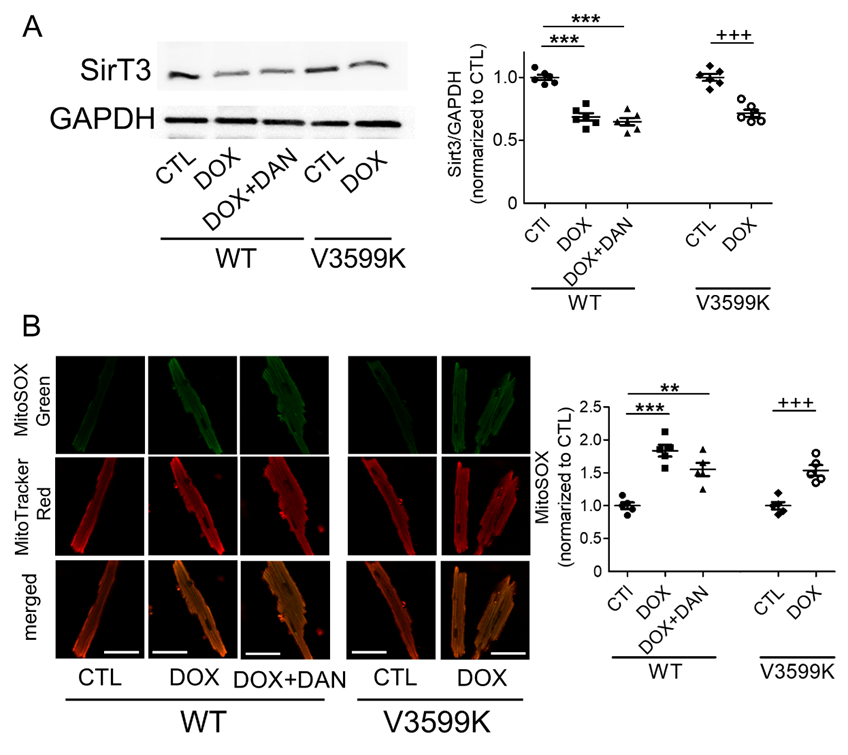


**Supplemental Figure 16. Evaluation of mitochondrial oxidative damage** Evaluation of mitochondria from mice under control conditions (CTL), at 5 days after DOX (6 mg/kg body weight) administration (DOX), and 5 days after DOX administration with oral DAN administration (DOX+DAN). (A) Representative images of Western blots of SirT3 and GAPDH (left), and summarized data (right). N = 6 hearts. (B) Representative fluorescence images of mitochondrial ROS detected by MitoSOX (green, upper panels) and Mito Tracker Red (red, middle panels) (left), and summarized data (right). Scale bar: 50 μm. N = 27–42 cells from 5 hearts. Values for individual mice are plotted with mean ± SEM. **P < 0.01, ***P < 0.001 (ANOVA with Tukey's post-hoc test). +++P < 0.001 (unpaired Student’s t test).

DAN = dantrolene; DOX = doxorubicin; ROS = reactive oxygen species; WT = wild-type.


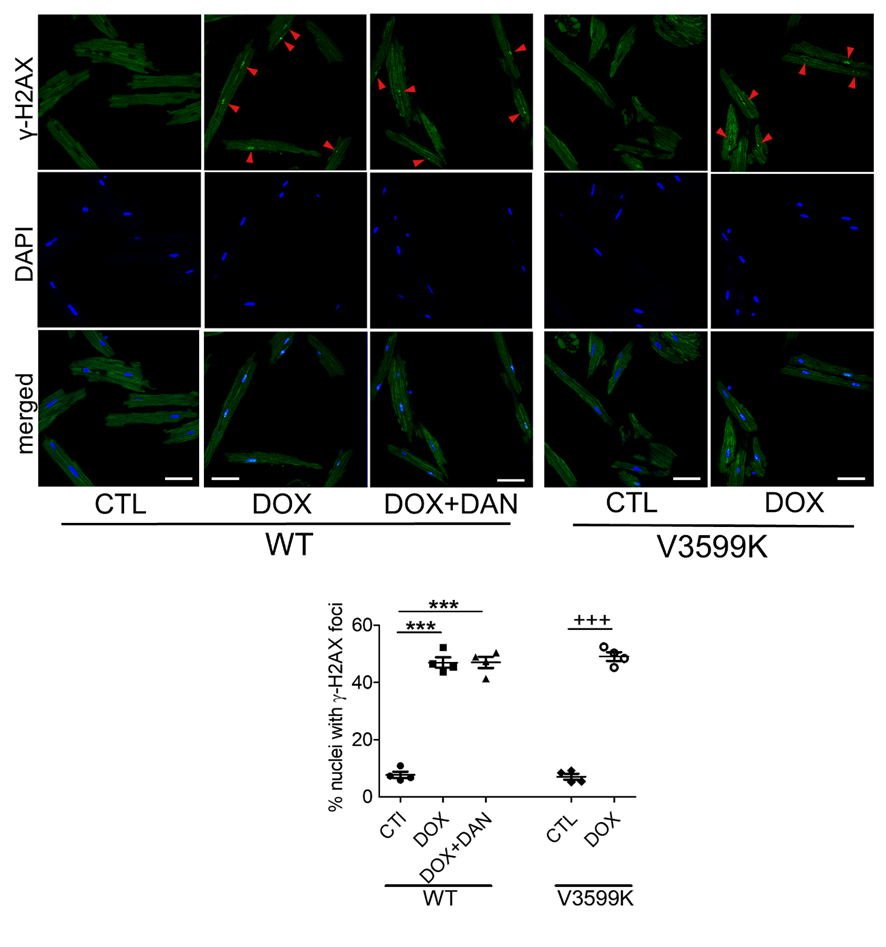


**Supplemental Figure 17. Evaluation of DNA damage** Evaluation of DNA double-strand breaks from mice under control conditions (CTL), at 5 days after DOX (6 mg/kg body weight) administration (DOX), and 5 days after DOX administration with oral DAN administration (DOX+DAN). Representative fluorescence images of γ-H2AX (upper panels) and DAPI(middle panels), and summarized data. Arrowheads indicate γ-H2AX ^+^ nuclei. Scale bar: 50 μm. (n = 22–38 cells from 4 hearts, Values for individual mice are plotted with mean ± SEM. ***P < 0.001 (ANOVA with Tukey's post-hoc test). +++P < 0.001 (unpaired Student’s t test).

DAN = dantrolene; DOX = doxorubicin; ROS = reactive oxygen species; WT = wild-type.

Data Availability

3-dimensional structure in Supplemental Figure 15 was referred from PDBID: 6JV2.^7^

**Supplemental References**

1. Delgado RM 3rd, Nawar MA, Zewail AM, et al. Cyclooxygenase-2 inhibitor treatment improves left ventricular function and mortality in a murine model of doxorubicin-induced heart failure. *Circulation.* 2004;109:1428-1433.
2. Neilan TG, Blake SL, Ichinose F, et al. Disruption of nitric oxide synthase 3 protects against the cardiac injury, dysfunction, and mortality induced by doxorubicin. *Circulation.* 2007;116:506-514.
3. Uchinoumi, H, Yano M, Suetomi T, et al. Catecholaminergic polymorphic ventricular tachycardia is caused by mutation-linked defective conformational regulation of the ryanodine receptor. *Circ Res.* 2010;106:1413-1424.
4. Sufu-Shimizu Y, Okuda S, Kato T, et al. Stabilizing cardiac ryanodine receptor prevents the development of cardiac dysfunction and lethal arrhythmia in Ca^2+^/calmodulin-dependent protein kinase II δ c transgenic mice. *Biochem Biophys Res Commun.* 2020;524:431-438.
5. Devereux RB, Alonso DR, Lutas EM, et al. Echocardiographic assessment of left ventricular hypertrophy: comparison to necropsy findings. *Am J Cardiol.* 1986;57:450-458.
6. Yamamoto T, Yano M, Xu X, et al. Identification of target domains of the cardiac ryanodine receptor to correct channel disorder in failing hearts. *Circulation.* 2008;117:762-72.
7. Gong D, Chi X, Wei J, et al. Modulation of cardiac ryanodine receptor 2 by calmodulin. *Nature.* 2019;572:347-351. https://doi.org/10.2210/pdb6jv2/pdb
